# Epigenetic activation of CD274/PD-L1 by the MSL complex expands its role beyond dosage compensation

**DOI:** 10.3389/fimmu.2025.1711451

**Published:** 2025-12-02

**Authors:** Aiping Wen, Xuanfei Feng, Yingying Li, Xueli Cui, Qixian Zou, Yong Cai, Jingji Jin, Yunxiao He

**Affiliations:** 1 Department of Gynecology and Obstetrics, Affiliated Hospital of North Sichuan Medical College, Nanchong, Sichuan, China; 2 Changchun GeneScience Pharmaceutical Co., Ltd., Changchun, Jilin, China; 3 School of Life Sciences, Jilin University, Changchun, Jilin, China

**Keywords:** histone acetyltransferase, CD274, transcriptional regulation, male-specific lethal, H4K16Ac

## Abstract

**Introduction:**

The regulation of CD274 (PD-L1), a pivotal immune checkpoint in cancer immunotherapy, remain incompletely understood. The male-specific lethal (MSL) complex, initially identified in *Drosophila* dosage compensation, contains the core subunit KAT8 (MOF), which catalyzes histone H4 lysine 16 acetylation (H4K16ac). However, whether the MSL complex directly regulates CD274 transcription has not been established.

**Methods:**

Using TIMER and GEPIA2, we charted pan-cancer expression of MSL subunits and their correlation with immune infiltration, integrating Kaplan–Meier survival and copy number variation (CNV) data to assess clinical relevance. CRISPR–Cas9 deletion of MSL1 or MSL3 in HEK293T cells, followed by RNA-seq, identified CD274 as a potential target. MSL1 knockdown or overexpression in LNCaP, HCT116, HeLa and MCF-7 cells confirmed regulation of CD274 protein, validated by rescue experiments in HEK293T cells. Luciferase reporter, ChIP–qPCR and ChIP–seq analyses collectively map the MSL-complex–CD274 regulatory axis.

**Results and discussion:**

Here we demonstrate that MSL1, a key subunit of the complex directly activates CD274 transcription by recruiting MOF to its promoter region and promoting H4K16 acetylation. Bioinformatic analyses revealed strong correlations between MSL1 expression, immune cell infiltration, and enrichment of immune-related gene sets across multiple cancer types. CRISPR/Cas9-mediated knockout of MSL1 or MSL3 markedly suppressed CD274 expression, whereas MSL1 overexpression enhanced CD274 levels and upregulated downstream immune- and apoptosis-related genes, including *BIRC3* and *HLA-A*. Dual-luciferase reporter assays, ChIP-qPCR and ChIP-seq further confirmed MSL1 binding near the –700 bp region of the CD274 promoter. Collectively, these findings uncover a previously unrecognized epigenetic mechanism linking the MSL complex to CD274 transcriptional regulation and identify MSL1 as a potential target for enhancing immunotherapy efficacy.

## 1 Introduction

Malignant tumors remain a major threat to human health, with both incidence and mortality continuing to rise globally. According to the World Health Organization (WHO), cancer is now the second leading cause of death worldwide (1). Despite significant advances in oncology, conventional treatment modalities—including surgery, radiotherapy, and chemotherapy—are still constrained by tumor heterogeneity, therapeutic resistance, and the risk of distant metastasis even after curative intervention (2–4). These limitations underscore an urgent need for innovative therapeutic strategies, which have become a major focus of modern cancer research.

Evasion of immune surveillance is a hallmark of cancer (5). Tumor cells employ multiple mechanisms to suppress antitumor immunity, thereby facilitating uncontrolled growth and dissemination. These mechanisms include downregulation of antigen-presenting molecules, secretion of immunosuppressive cytokines, and recruitment of immunosuppressive cells, all of which impair the recognition and cytotoxic activity of effector T cells (6). Over the past decade, immunotherapy has revolutionized by harnessing the patient’s own immune system to eliminate malignant cells. Among the most promising approaches are immune checkpoint inhibitors (ICIs) (7), chimeric antigen receptor T (CAR-T) cell therapy (8), and bispecific antibodies (bsAbs) (9). Clinically approved ICIs include antibodies targeting CD279 (PD-1) and CD274 (PD-L1) —such as pembrolizumab (Keytruda), nivolumab (Opdivo), and cemiplimab—as well as the anti-CTLA-4 antibody ipilimumab (10). CAR-T therapy has demonstrated remarkable efficacy in hematological malignancies, particularly in certain leukemias and lymphomas (11). Likewise, bispecific antibodies such as catumaxomab and blinatumomab have shown clinical benefit in patients with epithelial tumors and uveal melanoma (12). Despite these advances, immunotherapies remain limited by primary or acquired resistance, low response rates, and immune-related adverse events. These challenges highlight the critical need to elucidate the molecular mechanisms governing immune checkpoint regulation, thereby paving the way for more effective and durable therapeutic interventions.

Immune checkpoint molecules include CTLA-4, VISTA, TIM3, and PD-1 (CD279), among which CD279 and its ligand CD274 are the most extensively studied (13). CD279 is predominantly expressed on immune cells (14), where its engagement with ligands suppresses T cell activation and proliferation, thereby attenuating antitumor immunity (15). The interaction between CD279 on tumor-infiltrating lymphocytes and CD274 or CD273 (PD-L2) on tumor cells constitutes a major mechanism of immune evasion. Overexpression of CD279 in T cells reduces their proliferation, cytokine secretion, and cytotoxic activity (16). Under physiological conditions, CD274- CD279 binding maintains T cell homeostasis and prevents overactivation (17). However, within the tumor microenvironment, CD274 expressed on tumor cells engages CD279 on immune cells, leading to phosphorylation of immunoreceptor tyrosine motifs, recruitment of SHP1/2, and inhibition of ZAP70 and PI3K signaling. This signaling cascade ultimately impairs T cell effector function and promotes tumor immune escape (18). Consequently, the therapeutic blockade of CD279/CD274 axis has become a cornerstone of modern cancer immunotherapy.

The male-specific lethal (MSL) complex was first identified in *Drosophila melanogaster*, where it mediates dosage compensation to balance X-chromosome gene expression between sexes (19). In mammals, the MSL complex comprises four highly conserved subunits: MOF, MSL1, MSL2, and MSL3 (20). MSL1 functions as a scaffold protein that assembles the complex and promotes histone H4 acetylation at lysine 16 (21). Beyond its canonical role in dosage compensation, MSL1 regulates cell-cycle progression and proliferation, and can associate with autosomal promoters to modulate target gene transcription (22, 23). Increasing evidence indicates that MSL1 plays a regulatory role in tumor biology (24). For instance, DNA damage–induced alternative polyadenylation can elevate MSL1 expression through PCF11, thereby protecting cancer cells from apoptosis (25). In nasopharyngeal carcinoma, PBK-mediated phosphorylation of MSL1 enhances MSL complex occupancy at the *CD276* promoter, driving transcriptional activation of *CD276* and promoting immune evasion (26). However, the interaction between MSL1 and other immune regulatory factors remains poorly understood. Elucidating the mechanisms by which MSL1 regulates immune checkpoints may thus provide new insights into tumor immune regulation and identify potential therapeutic targets to improve patient outcomes. Bioinformatics analyses revealed that among MSL subunits, MSL1 displayed the most significant correlation with immune infiltration across multiple cancer types, with associated genes enriched in immune-related pathways. In MSL1- and MSL3-deficient cells, expression of the immune checkpoint gene *CD274* was markedly downregulated. Functional assays and chromatin immunoprecipitation (ChIP) analyses demonstrated that MSL1 binds to the *CD274* promoter and activates its transcription through MOF-mediated H4K16 acetylation. These findings uncover a previously unrecognized role of the MSL complex in regulating *CD274*, linking MSL1 to immune checkpoint control, apoptosis, and modulation of the tumor microenvironment, and highlighting it as a potential target to enhance tumor immunotherapy. Although this study establishes the transcriptional regulation of CD274 by the MSL1-MOF complex, the functional consequences—such as CD274 receptor binding, T cell co-culture, or apoptosis responses—remain to be explored.

## 2 Materials and methods

### 2.1 Antibodies

The following antibodies were used in this study: anti-MSL1 (mouse monoclonal, 24373-1-AP; Proteintech, Wuhan, China); anti-PD-L1/CD274 (rabbit monoclonal, A26086; ABclonal Technology, Wuhan, China); anti-MOF/KAT8/MYST1 (rabbit monoclonal, A3390; ABclonal Technology, Wuhan, China); and anti-MSL2 (rabbit polyclonal, ab83911; Abcam, Shanghai, China). Antibodies against NSL3, MSL3 and GAPDH (rabbit polyclonal) were generated in-house at Jilin University using bacterially expressed proteins. Polyinosinic-polycytidylic acid [Poly(I:C), P1530] was purchased from Sigma-Aldrich (Shanghai, China).

### 2.2 Cell culture

Human embryonic kidney (HEK293T), prostate carcinoma (LNCaP), colon carcinoma (HCT116), breast cancer (MCF-7), and cervical carcinoma (HeLa) cell lines were obtained from laboratory stocks. Cells were cultured in Dulbecco’s modified Eagle’s medium (DMEM, Meilunbio^®^, Dalian, China) supplemented with 10% fetal bovine serum (FBS, Procell, Wuhan, China) and 1% penicillin-streptomycin (P/S, Cytiva, Shanghai, China). Cultures were maintained at 37°C in a humidified atmosphere containing 5% CO_2_. Cell line identity was verified by short tandem repeat (STR) profiling within the past three years, and all experiments were conducted using mycoplasma-free cells.

### 2.3 Plasmid construction and transfection

The coding sequences of full-length MOF (NM_032188), MSL1 (NM_001012241), MSL2 (BC093790), MSL3 (BC031210), and CD274 (NM_014143.4) were subcloned into pcDNA3.1(–) expression vectors containing either Flag or Myc tags. Transient transfection was performed using polyethyleneimine (PEI, 23966, PolySciences, Beijing, China) according to the manufacturer’s instructions.

### 2.4 Generation of the CRISPR/Cas9-mediated human MSL1-KO cell line

Single-guide RNAs (gRNA) targeting exon 3 of hMSL1 and exon 2 of hMSL3 were designed using the CRISPR design tool (http:/crispr.mit.edu/) (27). The target sequences were 5’-ATGTCTCGGAAAGCTCCGGC-3’ for *MSL1* and 5’-ATCGTACAGCACTCGCGCCT-3’ for *MSL3*. The corresponding oligonucleotides were synthesized, annealed, and cloned into the pSpCas9 (BB)-2A-Puro (pX459, Addgene Cat#48139, Cambridge, MA, USA) vector via the *BbsI* restriction site to generate the knockout plasmids pX459-MSL1-KO and pX459-MSL3-KO. HEK293T cells at approximately 30% confluence were transfected with 2 μg of each plasmid using polyetherimide (PEI; Cat#23966, Poylsciences, Beijing, China) according to the manufacturer’s instructions. After 48 hours, cells were selected with 2 μg/mL puromycin to obtain single-cell colonies. Individual colonies were expanded in 96-well plates, and knockout efficiency was verified by Western blotting and Sanger sequencing of PCR-amplified target regions to confirm indel mutations.

### 2.5 shRNA knockdown

MSL1 knockdown was achieved using the pLVX-shRNA system in HEK293T, LNCaP, HCT116, and MCF-7 cells. The shRNA construct targeting MSL1 contained the sequence: GCACCGGACGTGTAGGAAAT.

### 2.6 Immunofluorescence staining

HEK293T cells with MSL1 or MSL3 knockout and HeLa cells were seeded onto coverslips placed in 24-well plates (NEST, 8D1007, Wuxi, China) and cultured to ~30% confluence. After 48 h, cells were fixed and subjected to immunofluorescence staining using antibodies against α-tubulin, MSL1, or CD274 followed by incubation with FITC or TRITC-conjugated secondary antibodies (1:300, Santa Cruz, sc-2012). Nuclei were counterstained with DAPI in Vectashield mounting medium (H-1200, Vector Laboratories, Burlingame, CA, USA). Fluorescent images were acquired an Olympus BX40F microscope equipped with a 40*×* silicon immersion objective (Olympus Corporation, Miyazaki, Japan).

### 2.7 Quantitative real-time PCR

Total RNA was isolated using RNAiso Plus (9109; Takara, Tokyo, Japan). For each sample, 1 µg of RNA was reverse transcribed into cDNA using the PrimeScript First Strand cDNA Synthesis Kit (6110A, Takara, Tokyo, Japan). Relative mRNA expression was measured with TB Green^®^ Premix Ex Taq ™ II (RR820A, Takara, Tokyo, Japan) on an Eco Real-Time PCR System (Illumina, Gene Company Limited, Hong Kong, China). Primers sequences are listed in **Table 1**.

**Table 1:** RT-qPCR and ChIP-qPCR primer sequences.

Gene name		Primer sequences
CD274	forward	5*′*-GCTGCACTAATTGTCTATTGGGA-3*′*
	reverse	5*′*-AATTCGCTTGTAGTCGGCACC-3*′*
MSL1	forward	5*′*-GCTTTCCGAGACATCCCAGAC-3*′*
	reverse	5*′*-GGCTCCTCAATTCACGTTTACAA-3*′*
MSL2	forward	5*′*-AGCATCCTAGTGAACTGCTACA-3*′*
	reverse	5*′*- TGAGGTTGAAGGTAAAGGGGAA-3*′*
MSL3	forward	5*′*-AACAGGAGGAAACGGTTAGTGA-3*′*
	reverse	5*′*-TGTGGCATAACGTGATGGTGA-3*′*
MOF	forward	5*′*-GGAAAGTCCCAGAACTGTAGATG-3*′*
	reverse	5*′*-AAGAGTAGGTTCAGGAGTTGGAA-3*′*
GAPDH	forward	5*′*-TGTGGGCATCAATGGATTTGG-3*′*
	reverse	5*′*-ACACCATGTATTCCGGGTCAAT-3*′*
BIRC3	forward	5*′*-AAGCTACCTCTCAGCCTACTTT-3*′*
	reverse	5*′*-CCACTGTTTTCTGTACCCGGA-3*′*
HLA-A	forward	5*′*-GACGCCCCCAAAACGCATA-3*′*
	reverse	5*′*-TGGGCAAACCCTCATGCTG-3*′*
STAT3	forward	5*′*-CAGCAGCTTGACACACGGTA-3*′*
	reverse	5*′*-AAACACCAAAGTGGCATGTGA-3*′*

### 2.8 Western blotting

HEK293T, LNCaP, HCT116, HeLa and MCF-7 cells were lysed in RIPA buffer supplemented with phosphatase and protease inhibitor cocktails. Proteins extracts were denatured at 97°C for 10 min, resolved by SDS–PAGE, and transferred onto poly(vinylidene difluoride) membrane (Millipore, Burlington, MA, USA). Membranes were blocked with 5% non-fat milk for 1 h, incubated with primary antibodies, and visualized using an ECL chemiluminescence kit (Thermo Fisher Scientific).

### 2.9 RNA-sequencing

Total RNA from MSL1/3-WT or MSL1/3-KO HEK293T cells was extracted using RNAiso Plus (Cat. No. 9109; Takara, Tokyo, Japan) and subsequently submitted to Biomarker Technologies Co., Ltd. (Beijing, China) for RNA-Seq analysis. Sequenced reads were aligned to the human GRCh38 reference genome using HISAT2 (v2.1.0) (28). Gene-level read counts were obtained using the SummarizeOverlaps function in the R package (https://www.r-project.org/). Differential gene expression analysis was performed with DESeq2 (29). Genes with a false discovery rate (FDR) < 0.01 and an absolute fold change ≥ 2 were considered significantly expressed.

### 2.10 Chromatin immunoprecipitation–sequencing and data analysis

ChIP-seq data for H4K5/8/12/16ac, H4K16ac and KAT8 (MOF) were obtained from GEO accessions GSE23730, GSE58953 and GSE96487, respectively. MSL1 and H4K16ac ChIP assays were performed in HEK293T cells. Sonicated chromatin (200–500 bp DNA fragment) was incubated with specific antibodies overnight at 4°C. After reverse cross-linking, DNA was extracted and ChIP-sequencing (ChIP-Seq) libraries were prepared using NEBNext Ultra DNA Library Prep Kit (E7370, NEB, USA) according to the manufacturer’s manual and were sequenced using Illumina NovaSeq 6000. ChIP-Seq raw reads were trimmed and filtered for quality and adaptors using fastp (30); subsequently, the reads were mapped to the human assembly GRCh38 using Bowtie2 (31) and were de-duplicated using MarkDuplicates in Picard Tools (v2.18.23). The genome-wide signal of ChIP data was normalized to input data by using bamCompare and bamCoverage from deepTools (v3.2.1) (32). Visualization of ChIP-Seq was performed using the Integrative Genomics Viewer (IGV) software (33).

### 2.11 Bioinformatics and database analysis

The expression levels of MSL complex subunits and their associations with immune infiltration across various were analyzed using the TIMER database (based on raw data from TCGA and GTEx). Kaplan–Meier survival curves were generated using GEPIA2, where TCGA tumor samples were divided into MSL1-high (top 50%) and MSL1-low (bottom 50%) groups according to the median expression level. The follow-up period was ≤ 120 months, and survival differences were evaluated using the log-rank test. Copy number alterations were defined by GISTIC 2.0 (hg19, q < 0.25) and categorized into five levels (from deep deletion to high amplification). TIMER 2.0 was used to extract purity-corrected immune infiltration abundance. Immune-related gene sets were obtained from GeneCards (Score ≥ 20) and subjected to functional enrichment analysis using Metascape (minimum overlap = 3, P < 0.01, minimum enrichment = 1.5) to construct a functional enrichment network. The expression of MSL subunits and their correlations with immune infiltration were analyzed across 32 cancer types. Among the three subunits of the MSL complex (MSL1, MSL2, and MSL3), those showing statistically significant correlations (p < 0.05) with cancer were included in the final statistical analysis. Gene Ontology (GO) enrichment analysis (FDR < 0.01) and Reactome pathway analysis were conducted with the GO and Reactome database using Metascape (34).

### 2.12 Chromatin immunoprecipitation assay

For each ChIP assay, 1 × 10^7^ HEK293T cells were crosslinked with 1% paraformaldehyde for 10 min at room temperature, followed by quenching with 125 mM glycine for 5 min. Cells were lysed and sonicated using an ultrasonic disruptor (Scientz Biotechnology, Ningbo, China) for 30 min (5 s on/5 s off cycles). MSL1 antibody was pre-coupled to Protein-A/G magnetic beads (Sigma-Aldrich) and incubated with cell lysates for 8 h at 4°C. Chromatin–DNA complexes were eluted with buffer for 15 min, and DNA fragments were analyzed by qPCR, with IgG as a negative control. Each experiment was independently repeated three times, and each sample was assayed in triplicate. The following primers were used: CD274 promoter (−1178 to −1117 bp), forward 5*′*-GCTGGGCCCAAACCCTATT-3*′* and reverse 5*′*-TTTGGCAGGAGCATGGAGTT-3*′*; CD274 promoter (−871 to −783 bp), forward 5*′*-AGAGCACCTAGAAGTTCAGCG-3*′* and reverse 5*′*-GGCCCAAGATGACAGACGAT-3*′*.

### 2.13 Statistical analysis

All data were derived from at least three independent experiments. Analyses were performed using SPSS v26 (IBM Corp., Armonk, NY, USA). Results are presented as mean *±* SD. Comparisons between two groups were assessed with an unpaired Student’s *t*-test, while differences among multiple groups were evaluated by one-way ANOVA. A p-value < 0.05 was considered statistically significant.

## 3 Results

### 3.1 Bioinformatics analyses reveal a potential link between the MSL complex and immune function

**Figure 1:**
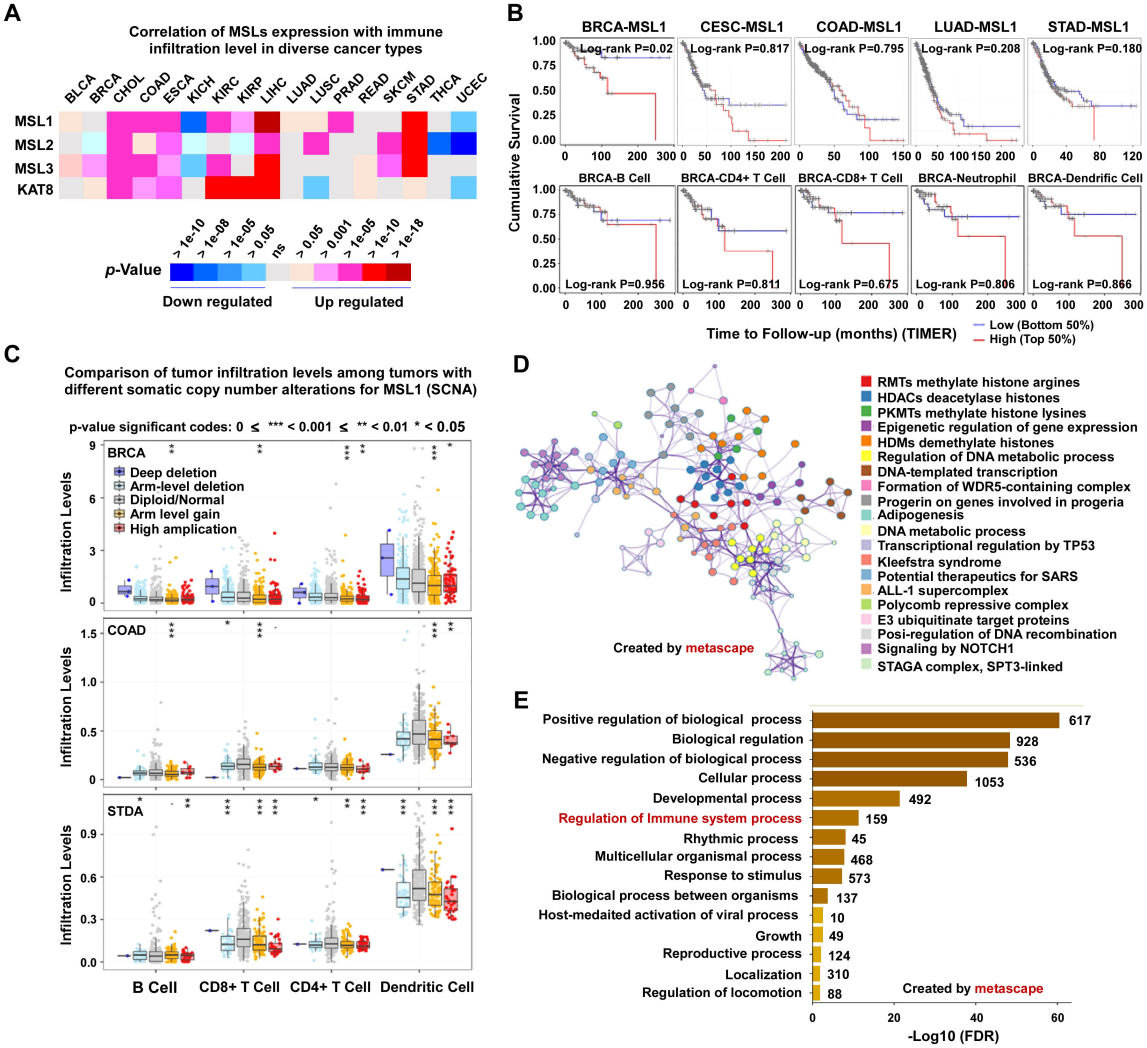
MSL1 is associated with immune infiltration and prognosis across cancers. **(A)** Heatmap showing correlations between MSL1/2/3 expression and immune infiltration across cancer types (TIMER database). **(B)** Kaplan-Meier curves of overall survival stratified by MSL1 expression in multiple cancers and immune cell subsets. **(C)** Box plots comparing immune infiltration levels in tumors with different somatic copy number alterations (SCNA) of MSL1. **(D)** Metascape network visualization of biological processes and molecular functions enriched among MSL1-associated genes. **(E)** Bar plot ranking enriched biological processes linked to MSL1 by statistical significance (–log10 FDR).

To investigate the relationship between the MSL complex and immune regulation, we first examined the expression profiles of MSL complex subunits across multiple tumor types using the TIMER database. Distinct expression patterns were observed among cancers (**Figure 1A**). Specifically, MSL subunits were significantly upregulated in several malignancies, including cholangiocarcinoma (CHOL), colon adenocarcinoma (COAD), esophageal carcinoma (ESCA), kidney renal clear cell carcinoma (KIRC), liver hepatocellular carcinoma (LIHC), and stomach adenocarcinoma (STAD), where their expression showed a strong positive association with immune cell infiltration (**Table 2**). In contrast, MSL complex expression was reduced in kidney chromophobe carcinoma (KICH) and uterine corpus endometrial carcinoma (UCEC), where it negatively correlated with immune infiltration (**Table 2**).

**Table 2:** Correlation between MSL1-3 expression and immune cell infiltration across cancer types (TIMER).

Tumor	MSL1 (*P*-value)	Regulated type	MSL2 (*P*-value)	Regulated type	MSL3 (*P*-value)	Regulated type	MYST1 (*P*-value)	Regulated type
BLCA	0.04492	Up	0.25662	ns	4.62E-03	Up	0.251081	ns
BRCA	0.14378	ns	1.42E-03	Down	5.32E-04	Up	0.042148	Up
CHOL	1.58E-08	Up	4.29E-08	Up	2.06E-09	Up	1.58E-08	Up
COAD	7.30E-08	Up	1.63E-03	Up	3.88E-09	Up	2.29E-04	Up
ESCA	4.03E-05	Up	6.76E-05	Up	2.69E-04	Up	6.69E-04	Up
KICH	7.71E-09	Down	9.55E-03	Down	2.82E-08	Down	0.596791	ns
KIRC	1.15E-05	Up	0.14252	ns	5.33E-09	Up	2.98E-13	Up
KIRP	2.72E-04	Up	7.62E-03	Down	0.34445	ns	6.19E-10	Up
LIHC	2.60E-19	Up	5.80E-08	Up	2.01E-11	Up	2.87E-13	Up
LUAD	2.48E-03	Up	0.08841	ns	0.823263	ns	0.354591	ns
LUSC	3.94E-03	Up	9.87E-09	Up	0.317808	ns	5.59E-06	Down
PRAD	1.18E-07	Up	0.33214	ns	0.109063	ns	0.265251	ns
READ	0.15319	ns	0.10251	ns	0.011912	Up	0.004779	Up
SKCM	0.10306	ns	6.80E-08	Up	1.18E-04	Up	0.127372	ns
STAD	5.89E-13	Up	8.52E-11	Up	2.12E-13	Up	0.001229	Up
THCA	0.25789	ns	3.06E-09	Down	0.141112	ns	0.454101	ns
UCEC	1.29E-07	Down	7.04E-12	Down	0.096973	ns	7.59E-06	Down

To assess the clinical relevance of MSL1 expression, Kaplan–Meier survival analyses were performed using GEPIA2 (**Figure 1B**). High MSL1 expression was significantly associated with poorer overall survival in several cancer types. Notably, in breast invasive carcinoma (BRCA), patients with high MSL1 expression exhibited significantly worse survival compared with those with low expression (log-rank *P* = 0.02). However, stratified analysis based on immune cell subtypes—including B cells, CD4^+^ T cells, CD8^+^ T cells, neutrophils, and dendritic cells—showed no significant association between MSL1 expression and survival in BRCA. These findings suggest that MSL1 may influence the tumor immune microenvironment, thereby potentially modulating immune evasion and therapeutic response.

To further explore the role of MSL1 in the tumor microenvironment, we examined the relationship between somatic copy number alterations (SCNA) of MSL1 and immune cell infiltration across cancers using TIMER 2.0 (**Figure 1C**). In BRCA, deep deletion of MSL1 was significantly associated with reduced infiltration of B cells, CD8^+^ T cells, and CD4^+^ T cells compared with the diploid/arm-level group (*p* < 0.05). Conversely, arm-level loss and high-level gain of MSL1 were associated with increased infiltration of CD4^+^ T cells and dendritic cells (*p* < 0.05 and *p* < 0.01, respectively). In COAD, arm-level loss of MSL1 correlated with reduced infiltration of B cells and CD8^+^ T cells (*p* < 0.05 and *p* < 0.01), whereas arm-level gain was linked to increased CD4^+^ T cell infiltration (*p* < 0.05). In STAD, arm-level gain of MSL1 was significantly correlated with increased dendritic cells infiltration (*p* < 0.05). Collectively, these findings indicate that MSL1 SCNAs may contribute to tumor immune evasion by modulating immune cell infiltration within the tumor microenvironment.

Finally, to characterize the biological processes associated with MSL1, we identified MSL1-related gene sets from the GeneCards database and constructed a gene regulatory network using Metascape (**Figure 1D**). MSL1 expression was significantly correlated with multiple biological processes, including histones arginine methylation by PRMTs, histone deacetylation by HDACs, epigenetic regulation of gene expression, and several disease-associated pathways. Gene Ontology (GO) enrichment analysis further revealed strong enrichment in processes such as regulation of biological activity, response to stimuli, cellular localization, and development (**Figure 1E**). Importantly, immune-related pathways were also significantly enriched, further supporting a role for MSL1 in regulating immune responses. Together, these findings suggest a previously unrecognized association between MSL1 and CD274 (PD-L1), highlighting a potential epigenetic link between the MSL complex and tumor immune regulation.

### 3.2 MSL1/MSL3 deficiency links CD274 expression to immune-related genes across tumors

**Figure 2:**
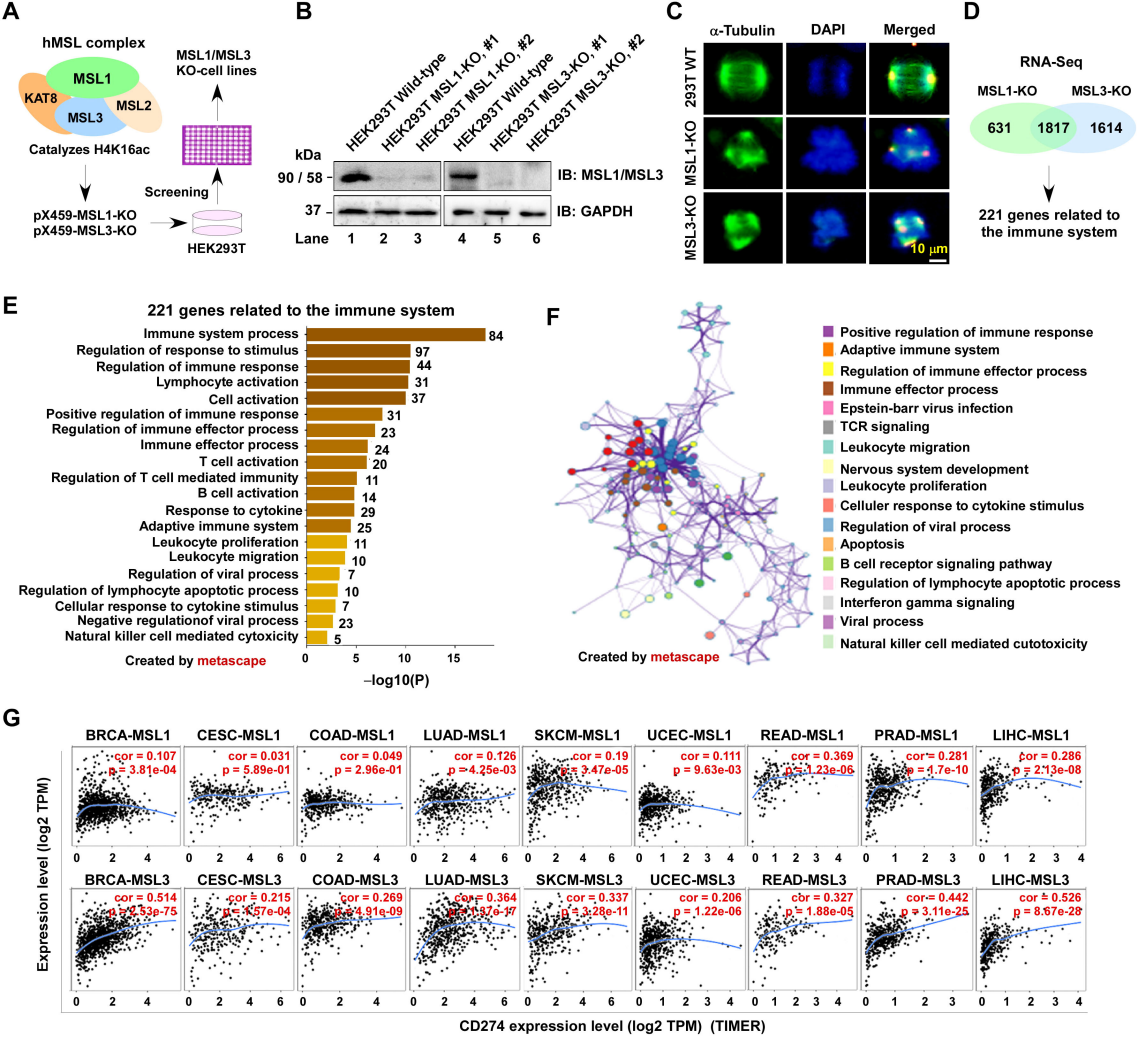
Functional characterization of MSL1 and MSL3 knockout cell lines. **(A)** Generation of knockout models. Schematic representation of MSL1- and MSL3-deficient cell lines generated by CRISPR/Cas9. **(B)** Validation of knockout efficiency. Western blot analysis confirming the loss of MSL1 and MSL3 protein expression in respective knockout cell lines. **(C)** Disruption of microtubule organization. Immunofluorescence images showing α-tubulin (green) and nuclei (DAPI, blue) in control and knockout cells. Merged panels highlight microtubule disorganization upon MSL1/3 depletion. Scale bar: 10 μm. **(D)** Shared immune-related gene sets. Venn diagram showing 221 immune-related DEGs common to MSL1- and MSL3-knockout cells. **(E)** Enriched biological processes. Bar plot of significantly enriched GO terms among the 221 shared DEGs identified by Metascape; bar length indicates –log10 (FDR). **(F)** Functional interaction networks. Metascape network visualization of immune -related biological processes among the shared DEGs, with nodes color-coded by functional category. **(G)** Correlation with CD274 expression. Scatter plots showing Pearson correlation between MSL1 or MSL3 and CD274 expression across cancer types. Upper, MSL1 vs CD274; lower, MSL3 vs CD274. Correlation coefficients (cor) and P values are shown.

We generated MSL1- and MSL3-knockout (KO) HEK293T (embryonic kidney 293T) cell lines using the CRISPR/Cas9 system (**Figure 2A**). Western blot analysis confirmed efficient depletion of MSL1 and MSL3 proteins compared with wild-type controls (**Figure 2B**). Immunofluorescence analysis further revealed marked alterations in microtubule organization in MSL1- and MSL3-KO cells relative to wild-type cells (**Figure 2C**).

To explore the functional consequences of MSL1 and MSL3 loss, RNA-Seq was performed on the KO cell lines. Compared with controls, 2,448 differentially expressed genes (DEGs) were identified in MSL1-KO cells and 3,431 DEGs in the MSL3-KO cells, with 1,817 shared between the two groups. Among these, 221 genes were classified as immune-related (**Figure 2D**). GO enrichment analysis (**Figure 2E**) revealed significant enrichment in pathways related to immune system process, immune response, lymphocyte activation, cytokine-mediated signaling, immune response, and T cell and B cell activation. Network analysis further highlighted processes associated with adaptive immunity, immune effector functions, and antiviral responses (**Figure 2F**). These findings suggest that MSL1 and MSL3 may directly or indirectly modulate immune-related transcriptional programs.

Notably, among the DEGs, CD274 (encoding PD-L1) emerged as one of the most prominently downregulated genes. In tumor cells, CD274 interacts with CD279 on T cells, leading to phosphorylation of immunoreceptor tyrosine motifs, suppression of T cell signaling, and subsequent immune evasion (35). We therefore hypothesize that CD274 may represent a downstream target of MSL1. To test this possibility in a broader oncogenic context, we analyzed the correlation between MSL1, MSL3, and CD274 expression across multiple cancer types using the TIMER2.0 database. Significant correlations were observed, with Spearman coefficients ranging from 0.031 to 0.526 (all *p* < 0.05; **Figure 2G**), supporting the notion that MSL1 and MSL3 cooperatively regulate CD274 expression and contribute to tumor immune escape.

### 3.3 MSL1 positively regulates CD274 expression across cancer cell lines

**Figure 3:**
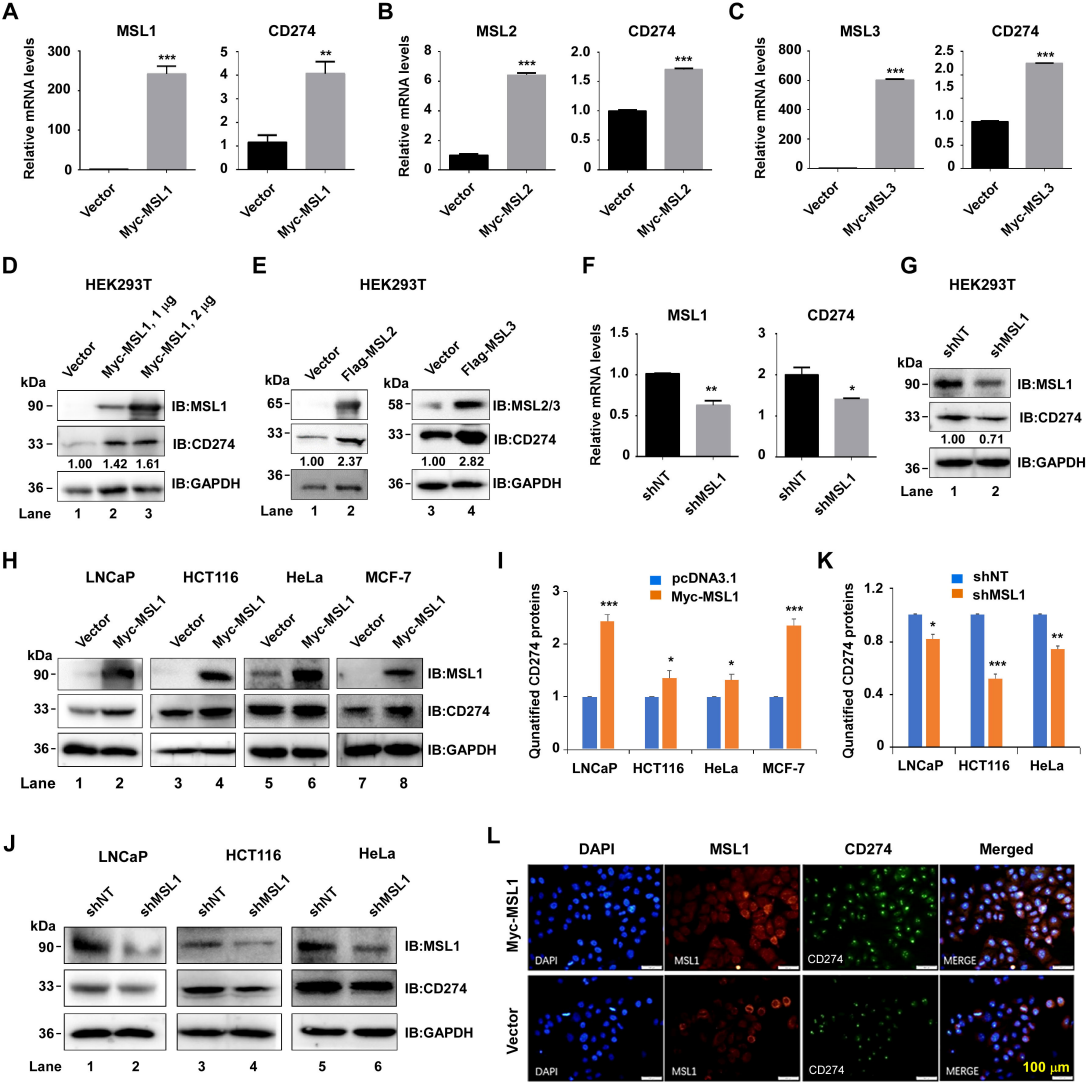
MSL1 and MSL3 regulate CD274 expression across multiple cell models. **(A–C)**. Overexpression assays. qRT-PCR analysis showing MSL1, MSL2, and MSL3 (left) and corresponding CD274 (right) mRNA levels following transfection with Myc-MSL1, Myc-MSL2, and Myc-MSL3. **(D, E)**. Protein induction by overexpression Western blot analysis of MSL1 **(D)**, MSL2 (**E**, left) or MSL3 (**E**, right) and CD274 in HEK293T cells transfected with Myc-MSL1 and Flag-tagged MSL2 or MSL3 plasmids. **(F, G)**. Knockdown assays. qRT-PCR **(F)** and western blot **(G)** showing reduced MSL1 and CD274 expression upon shRNA-mediated MSL1 silencing. **(H)**. Overexpression across cancer cell lines. MSL1 and CD274 protein expression of MSL1 and CD274 in LNCaP, HCT116, HeLa, and MCF-7 cell lines following transfection with Myc-MSL1 plasmid. Quantification of CD274 protein expression corresponding to **I**. **(J)** Western blot analysis of MSL1 and CD274 protein levels in LNCaP, HCT116, and HeLa cells after shMSL1 transfection. Quantification of CD274 protein expression corresponding to **K**. **(L)** Co-localization analysis. Immunofluorescence of HeLa cells showing MSL1 (red) and CD274 (green) co-localization following Myc-MSL1 overexpression; nuclei are stained with DAPI (blue). Scale bar, 100 µm. Statistical significance: *p < 0.05; **p < 0.01; ***p < 0.001. Data are presented as mean ± SEM (standard error of the mean) from three independent experiments (n=3). GAPDH served as control in all Western blot analyses.

We next examined the effects of MSL1, MSL2, and MSL3 on CD274 expression. Transient transfection of HEK293T cells with Myc-MSL1, Myc-MSL2, or Myc-MSL3 plasmids significantly increased CD274 mRNA levels by approximately 4.12-, 1.45-, and 2.61-fold, respectively, as determined by RT-qPCR (**Figures 3A–C**). Increasing doses of Myc-MSL1 plasmid (0, 1, and 2 μg) induced a dose-dependent elevation of CD274 protein levels (**Figure 3D**). Similarly, overexpression of Flag-MSL2 or Flag-MSL3 enhanced CD274 protein expression (**Figure 3E**). To validate this regulatory relationship, we performed shRNA-mediated knockdown of MSL1. Transient transfection of HEK293T cells with pLVX-ZsGreen-shMSL1 markedly reduced MSL1 expression, which was accompanied by a significant decrease in CD274 mRNA compared with shNT controls (**Figure 3F**). Consistently, Western blot analysis confirmed that MSL1 silencing diminished CD274 protein levels (**Figure 3G**).

Given the robust effects of MSL1 in HEK293T cells, we further evaluated its role in multiple cancer cell lines. Transient overexpression of Myc-MSL1 in LNCaP (prostate cancer), HCT116 (colon cancer), MCF-7 (breast cancer), and HeLa (cervical cancer) cells consistently elevated CD274 protein levels, as shown by Western blotting (**Figure 3H**, quantification in **Figure 3I**). Conversely, MSL1 knockdown with pLVX-ZsGreen-shMSL1 reduced CD274 protein abundance by 18% in LNCaP, 47% in HCT116, and 26% in HeLa cells (**Figure 3J**, quantification in **Figure 3K**). Immunofluorescence analysis in HeLa cells further revealed that MSL1 predominantly localized to the cytoplasm, whereas CD274 was distributed in both the cytoplasm and nucleus. Importantly, MSL1 overexpression enhanced CD274 fluorescence intensity (**Figure 3L**).

Collectively, these findings demonstrate that MSL1 acts as a positive regulator of CD274 expression across diverse cancer cell lines, as evidenced by reciprocal and consistent results from both gain- and loss-of-function experiments.

### 3.4 MSL1 modulates upstream and downstream regulators of CD274 expression

**Figure 4:**
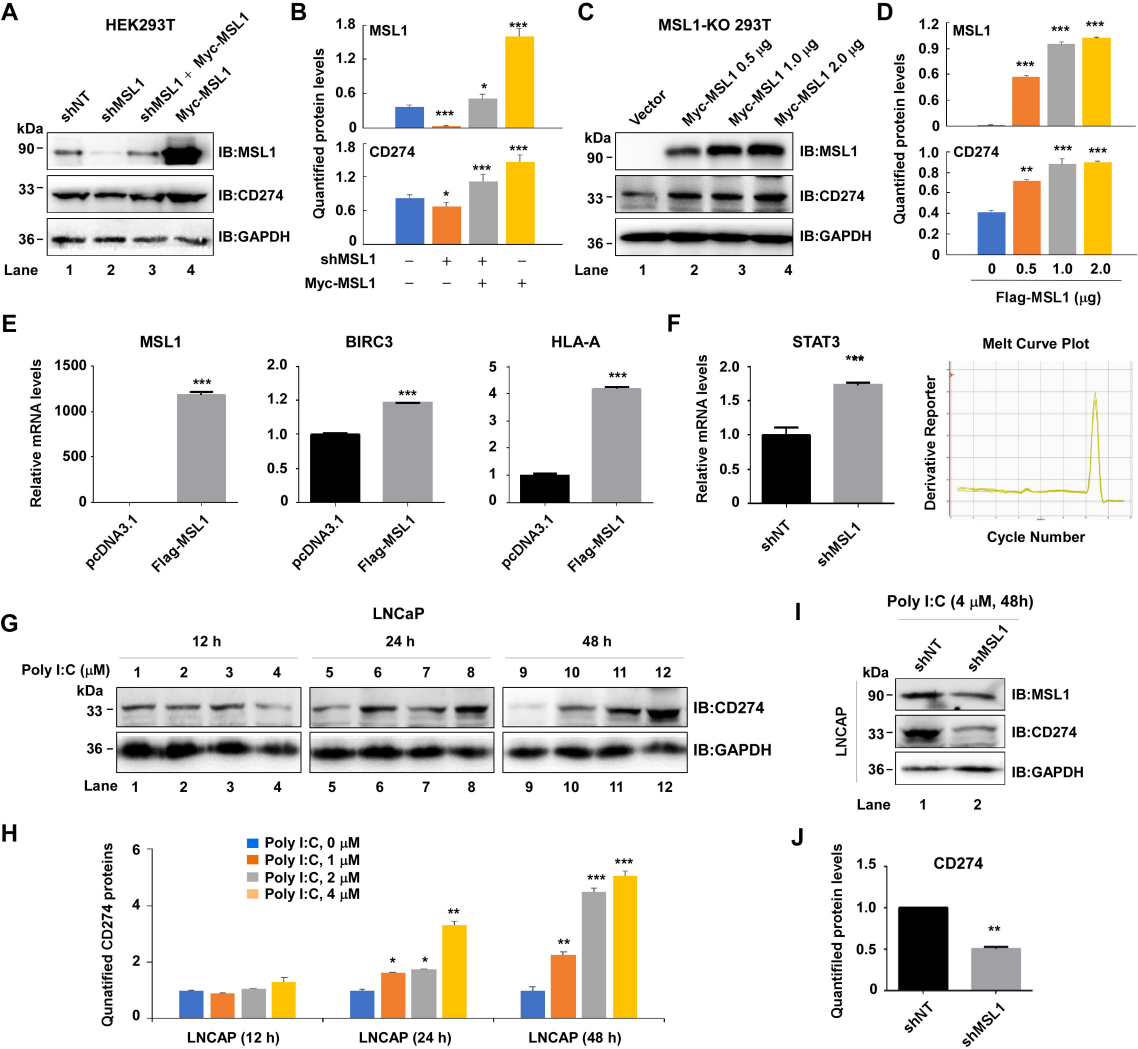
Regulation of CD274 expression by MSL1. **(A, B)**. Western blot Analysis **(A)** and quantification **(B)** of MSL1 and CD274 protein levels in HRK293T cells transfected with shNT (lane 1), shMSL1 (lane 2), shMSL1 followed by Myc-MSL1 (lane 3), or Myc-MSL1 alone (lane 4). **(C, D)**. Western blot analysis **(C)** and quantification **(D)** of MSL1 and CD274 expression in HEK293T cells transfected with increasing doses of Myc-MSL1 plasmid. **(E)** Quantitative PCR analysis of MSL1, BIRC3, and HLA-A mRNA expression following Flag-MSL1 overexpression. **(F)** STAT3 mRNA expression in HEK293T cells after shMSL1 transfection (left) and validation of STAT3 primer amplification curve (right). **(G, H)**. Western blot analysis **(G)** and quantification **(H)** of CD274 expression in LNCaP cells treated with Poly I:C at different concentrations for the indicated times. **(I, J)**. Western blot analysis **(I)** and quantification **(J)** of MSL1 and CD274 expression in LNCaP cells treated with Poly I:C (4 μM, 48 h). Statistical significance: *p < 0.05; **p < 0.01; ***p < 0.001. Data are presented as mean ± SEM from three independent experiments (n=3). GAPDH served as loading control in all Western blot analyses.

To further confirm the regulatory role of MSL1 in CD274 expression, we conducted rescue experiments. HEK293T cells were first transfected with pLVX-ZsGreen-shMSL1 or control shNT, followed 48 h later by transient complementation with Myc-MSL1. As shown in **Figure 4A**, MSL1 knockdown reduced CD274 protein levels, whereas reintroduction of Myc-MSL1 restored CD274 expression (quantified in **Figure 4B**). Similarly, graded re-expression of MSL1 (0, 0.5, 1, and 2 μg) in MSL1-KO cells induced a dose-dependent increase in CD274 protein levels, with significant restoration observed even at 0.5 μg (**Figure 4C**, quantified in **Figure 4D**). These findings suggest that MSL1 deletion may active compensatory transcriptional programs, rendering CD274 expression highly responsive to MSL1 reintroduction, potentially through additional regulatory factors or pathways.

CD274 expression is known to be modulated by interferon signaling (36–38), the JAK–STAT pathway (39), and other transcriptional regulators. In addition, CD274 deletion downregulates genes involved in NF-κB signaling and antigen presentation. To determine whether MSL1 influences these pathways, we analyzed key downstream effectors using RT-qPCR. Overexpression of MSL1 significantly increased BIRC3, an anti-apoptotic factor, and HLA-A, a class I HLA molecule associated with antigen presentation (**Figure 4E**), suggesting that MSL1 modulates CD274-dependent downstream signaling, potentially affecting apoptosis and the tumor microenvironment. Interestingly, STAT3, a known CD274 promoter-binding transcription factor (40), was upregulated upon MSL1 knockdown (**Figure 4F**), implying a possible competitive or compensatory relationship between MSL1 and STAT3 in regulating CD274 transcription.

CD274 expression varies substantially among tumor types (**Figure 1A**). For example, it is expressed at low levels in prostate cancer, which may contribute to the poor response rates to immune checkpoint blockade therapy (41). Combination therapies with conventional anticancer agents can improve clinical outcomes, increasing response rates by up to 32%. Poly I:C, a synthetic double-stranded RNA analog, activates Toll-like receptor 3 (TLR3) signaling and enhances interferon responses (42); however, its impact on CD274 expression in prostate cancer remains unclear. Treatment of LNCaP cells with increasing concentrations of Poly I:C (1–4 μM) for 24–48 h upregulated CD274 protein levels, with maximal induction observed at 4 μM after 48 h, whereas shorter exposure (12 h) had no detectable effect (**Figure 4G**, quantified in **Figure 4H**).

To test whether MSL1 mediated this effect, LNCaP cells transfected with shMSL1 were treated with 4 μM Poly I:C for 48 h. Immunoblotting revealed a marked attenuation of Poly I:C–induced CD274 upregulation compared with control cells (**Figure 4I**, quantification in **Figure 4J**), indicating that MSL1 contributes to poly I:C-mediated CD274 induction, at least in part through the TLR3 signaling pathway.

### 3.5 MSL1 transcriptionally activates CD274 via a specific promoter region

**Figure 5:**
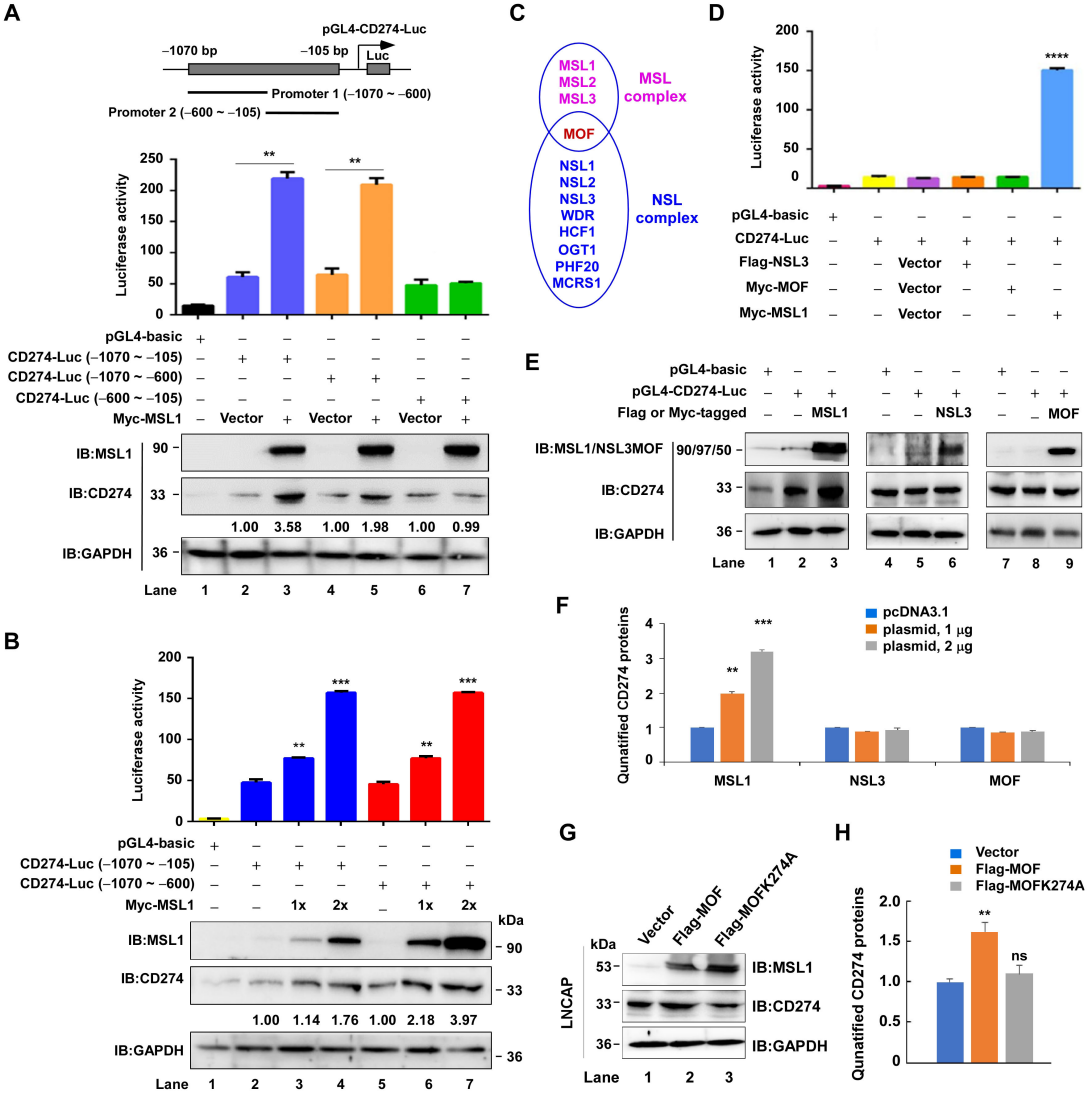
MSL1 transcriptionally regulates CD274 promoter activity. **(A)** Dual-luciferase reporter and Western blot analysis demonstrating the transcriptional regulation of CD274 by MSL1. Schematic shows luciferase reporter constructs containing CD274 promoter fragments (−1070 to −105 bp, −1070 to −600 bp, and −600 to −105bp) fused to firefly luciferase (Luc). **(B)** Luciferase reporter and Western blot analysis of CD274 promoter activity in cells co-transfected with pGL4-basic or pGL4-Luc constructs containing the proximal (−1070 to −105 bp) or distal (−1070 to −600 bp) CD274 promoter regions, together with increasing doses of Myc-MSL1 expression vector (1× or 2×). **(C)** Schematic illustration of MOF within two distinct complexes: MSL (left) and NSL (right). **(D)** Luciferase reporter assay assessing the impact of NLS3, MOF and MSL1 on CD274 promoter activation. Cells were co-transfected with the indicated plasmid combinations. **(E, F)**. Western blot analysis **(E)** and quantification **(F)** of CD274 protein levels in HEK293T cells following transfection with varying doses of Flag- or Myc-tagged MSL1, NSL3, or MOF. **(G, H)**. Western blot analysis **(G)** and quantification **(H)** of CD274 protein levels in LNCaP cells following transfection with Flag-MOF or Flag-MOFK274A plasmid. Statistical significance: **p < 0.01; ***p < 0.001; ****p < 0.0001. Data are presented as mean ± SEM from three independent experiments (n=3). GAPDH was used as the internal control in all Western blot analyses.

The above findings suggest that MSL1 regulates CD274 expression. To determine whether MSL1 directly promotes CD274 transcription, we performed dual-luciferase reporter assays. A CD274 promoter fragment (–1070 to –105 bp) was cloned into the pGL4.11 [luc2p] vector. To map the functional promoter region, three constructs were generated: pGL4-CD274-Luc (–1070 to –105bp), pGL4-CD274-Luc (–1070 to –600 bp), and pGL4-CD274-Luc (–600 to –105 bp). Each construct was co-transfected with Myc-MSL1 into HEK293T cells, and luciferase activity was measured 48 h later (**Figure 5A**, upper panel). MSL1 significantly increased luciferase activity driven by the –1070 to –600 bp fragment, reaching levels comparable to those of the full –1070 to –105 bp construct, whereas the –600 to –105 bp fragment showed no significant activation (**Figure 5A**, middle panel). To further validate this effect, HEK293T cells were transfected with increasing amounts of Myc-MSL1 (0, 1, and 2 μg) together with either the –1070 to –105 bp or –1070 to –600 bp reporter. Both constructs exhibited a dose-dependent increase in luciferase activity (**Figure 5B**, upper panel). Consistently, immunoblotting confirmed that MSL1 overexpression induced CD274 protein expression under these conditions (**Figures 5A, B**, lower panels). Collectively, these results demonstrate that MSL1 transcriptionally activates CD274 through the –1070 to –600 bp promoter region.

In human cells, MOF (KAT8) serves as the catalytic subunit of both the MSL and NSL complexes (**Figure 5C**). To examine whether MOF or NSL complex components also regulate CD274 transcription, HEK293T cells were co-transfected with Myc-MSL1, Myc-MOF, or Flag-NSL3 together with the–1070 to –105 bp CD274 promoter construct. Unlike MSL1, neither MOF nor NSL3 significantly altered luciferase activity (**Figure 5D**). Consistently, immunoblotting confirmed that overexpression of MOF or NSL3 did not affect CD274 protein levels (**Figure 5E**, quantification in **Figure 5F**). Together, these findings indicate that transcriptional activation of CD274 is specifically mediated by MSL1, and not by MOF and NSL3, highlighting a unique regulatory function of MSL1 within the MSL complex.

To further confirm that MSL1 functions as a complex in cells, we investigated the regulation of CD274 protein expression following transient transfection of wild-type (Flag-MOF) and catalytically inactive MOF plasmids (Flag-MOFK274A) in LNCaP cells. As anticipated, wild-type MOF, but not the mutant MOF, significantly enhanced CD274 protein expression (p<0.01) (**Figure 5G**, quantification in **Figure 5H**). These results confirm that CD274 expression is indeed driven by the MSL-MOF complex, highlighting its role in the regulation of CD274.

### 3.6 MSL1 binds to the CD274 promoter and recruits MOF-dependent acetylation

**Figure 6:**
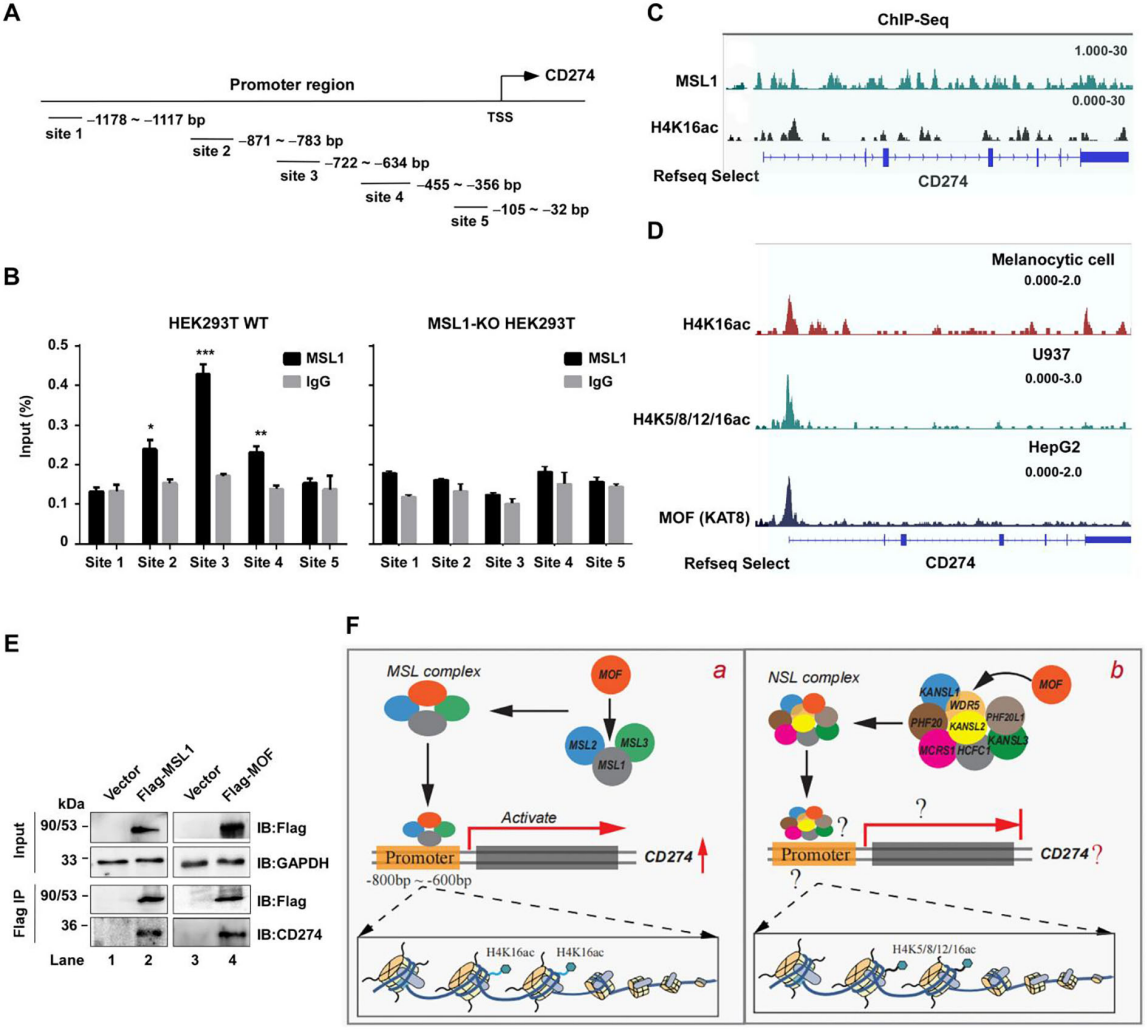
MSL1 binding and epigenetic regulation of the CD274 promoter. **(A)** CD274 promoter architecture. Schematic representation of the CD274 promoter region highlighting five putative binding sites. **(B)** MSL1 occupancy at the CD274 promoter. ChIP–qPCR analysis of MSL1 enrichment across the CD274 promoter in HEK293T cells. The left panel shows wild-type (WT) cells, whereas the right panel depicts MSL1- KO cells. Data are shows as mean ± SD from six independent experiments (n=6). Statistical significance: *p* < 0.05; *p* < 0.01; *p* < 0.001. **(C)** Epigenetic landscape of the CD274 locus showing MSL1 and H4K16ac enrichment in HEK293T cells. **(D)** Epigenetic landscape of the CD274 locus. IGV snapshots showing histone acetylation and MOF enrichment across the CD274 gene region in Melanocytic cells (H4K16ac, top), U937 cells (H4K5/8/12/16ac, middle), and HepG2 cells (KAT8/MOF, bottom). **(E)** Co-immunoprecipitation assays were performed in HEK293T cells using Flag-MSL1 and Flag-MOF constructs with an anti-Flag antibody. GAPDH was used as the internal control in all Western blot analyses. **(F)** Model of MSL and NSL complex–mediated regulation. Proposed mechanisms of CD274 transcriptional regulation. **(a)** The MSL complex activates CD274 transcription by recruiting MSL1 to the −800 to −600 bp region of the promoter, where MOF acetylates H4K16. **(b)** The NSL complex may also target the CD274 promoter through an unidentified subunit, potentially involving histone modifications such as H4K5/8/12/16ac. Gashed lines and question marks denote hypothetical or unresolved mechanisms.

To further explore whether MSL1 directly regulates CD274 transcription, we performed chromatin immunoprecipitation (ChIP)–qPCR assays using five primer sets spanning the CD274 promoter region (–1178 to –32 bp; **Figure 6A**). In wild-type HEK293T cells, anti-MSL1 ChIP demonstrated significant enrichment at Site 2 (–871 to –783 bp), Site 3 (–722 to –634 bp), and Site 4 (–455 to –356 bp), with the strongest binding observed at Site 3 (**Figure 6B**, left). This enrichment was completely abolished in MSL1-KO cells (**Figure 6B**, right), confirming that MSL1 binds to the CD274 promoter around –700 bp, thereby promoting transcriptional activation. To further confirm that MSL1 recruitment to the CD274 promoter region mediates H4K16ac deposition and promotes transcriptional activation of CD274, we performed ChIP-seq analyses for MSL1 and H4K16ac in HEK293T cells (**Figure 6C**). The overlapping enrichment peaks near the promoter region suggest that the MSL complex binds to chromatin through MSL1 to transcriptionally regulate CD274 expression. To validate this observation, we utilized public ChIP-seq datasets to examine the distribution of MOF (KAT8) and H4K16ac marks at the CD274 promoter (**Figure 6D**). Similarly, ChIP–seq analyses in melanocytes, U937, and HepG2 cells revealed high chromatin accessibility, accompanied by prominent enrichment of H4K16ac, H4K5/8/12/16ac, and KAT8 (MOF) across the CD274 promoter region. These findings indicate that MOF-mediated histone acetylation enhances promoter accessibility at the CD274 locus. Furthermore, co-immunoprecipitation assays confirmed the physical interaction between the MSL1 and MOF, providing additional evidence for their cooperative role in transcriptional regulation (**Figure 6E**). Collectively, we propose a model in which MSL1 directly binds to the CD274 promoter and recruits MOF to catalyze H4K16 acetylation, thereby promoting transcriptional activation (**Figure 6F**).

## 4 Discussion

CD279 (PD-1) and CD274 (PD-L1) are central immune checkpoint molecules targeted in cancer immunotherapy. Their signaling maintains immune homeostasis and peripheral tolerance but also promotes tumor progression and immune evasion by suppressing both innate and adaptive immune responses (43). Despite their clinical relevance, the mechanisms controlling CD279/CD274 expression remain incompletely understood. Elucidating these pathways may reveal new avenues for therapeutic intervention.

Epigenetic modifications play a pivotal role in regulating CD274 expression. Promoter methylation inversely correlates with CD274 levels across several cancers in gastric cancer (44), diffuse low-grade glioma (45), and non-small cell lung cancer (46), hypermethylation suppresses CD274 transcription, whereas hypomethylation enhances expression and immunosuppressive activity (47). Histone acetylation also contributes to CD274 induction. In breast cancer, TET2 recruits HDAC1/2 to remove H3K27ac at the CD274 promoter, thereby repressing its transcription (48). The MSL complex, which catalyzes histone H4K16 acetylation via its enzymatic subunit MOF, is another key transcriptional regulator. While its subunits (MSL1, MSL2, MSL3, and MOF) have distinct functions, MSL1 is notable for binding gene promoters and coordinating transcriptional activation. In nasopharyngeal carcinoma, PBK-mediated phosphorylation of MSL1 enhances CD276 transcription and facilitate immune evasion (26), implicating the MSL complex in tumor-associated immune regulation.

Here, we extended these findings by demonstrating that MSL1 is also involved in regulating another checkpoint molecule, CD274 (PD-L1). Bioinformatics analyses revealed strong correlations between MSL subunit expression and immune cell infiltration across multiple tumor types, with distinct patterns observed under different copy number variations. GeneCards enrichment analysis further indicated that MSL1-associated genes are enriched in immune-related pathways, supporting the notion that MSL1 contributes to shaping the tumor immune microenvironment. Unlike the previously reported phosphorylation-dependent mechanism, our data highlight an acetylation-associated regulatory pathway, in which MSL1 cooperates with MOF to promote H4K16ac-dependent transcriptional activation of CD274. This acetylation-based mechanism expands the functional spectrum of the MSL complex in immune regulation and provides new insights into how tumors exploit epigenetic modulators to evade immune surveillance. Collectively, these results identify MSL1 as a key epigenetic regulator of CD247 expression and a potential therapeutic target for enhancing the efficacy of cancer immunotherapy.

Beyond epigenetic regulation, multiple miRNAs (e.g., miR-34a, miR-138-5p) (49, 50) and transcription factors (HIF-1 α, STAT, NF-κB, YY1) (39, 51) are known to modulate CD274 expression. Consistently, RNA-seq analysis of MSL1- and MSL3-knockout cells revealed marked downregulation of CD274, along with reduced expression of genes involved in immune response and T cell signaling. Functional assays further confirmed the positive regulatory role of MSL1: its overexpression increased the expression of downstream targets such as BIRC3, an apoptosis regulator (36–38), and HLA-A, a key MHC-1 molecule (39), whereas MSL1 knockdown upregulated STAT3, a known CD274 regulator. Together, these results indicate that MSL1 modulates tumor immunity by influencing CD274 expression and its associated signaling networks.

MSL1 and MSL3 are essential for MSL complex assembly and chromatin targeting (52, 53). The catalytic subunit MOF drives H4K16 acetylation but also functions within the NSL complex (54). Although both complexes target H4K16, the NSL complex also targets H4K5 and H4K8, thereby enhancing chromatin accessibility and promoting transcription of housekeeping genes (54). Functionally, however, the two complexes diverge: the NSL complex supports cell proliferation, whereas the MSL complex lacks this activity. Our luciferase assays demonstrated that MOF and NSL3 alone do not activate CD274 transcription, whereas MSL1 does. Consistently, ChIP-seq analyses confirmed co-enrichment of MOF and H4K16ac at the CD274 locus. Signals of H4K5/8/12/16ac further suggest possible NSL occupancy, likely mediated by subunits other than NSL3.

Our earlier work showed that the MSL complex primarily regulates genes involved in cell growth and maintenance, whereas the NSL complex is linked to phosphoinositol-mediated signaling (55). Interestingly, these complexes may exert opposing effects: loss of MSL activity promotes epithelial-mesenchymal transition (EMT) and metastasis, whereas NSL activity appears to restrain proliferation and EMT. Based on our findings, we propose that MSL1 recruits MOF to the CD274 promoter to promote transcription through H4K16 acetylation. In contrast, when MOF associates with the NSL complex, it may exert distinct—possibly inhibitory—effects on CD274. Identifying the NSL subunits responsible for promoter occupancy on CD274 will be an important direction for future investigation.

In summary, this study uncovers a previously unrecognized role of the MSL complex in regulating the immune checkpoint molecule CD274. We show that MSL1 directly binds the CD274 promoter, recruits MOF, and promotes transcription through H4K16 acetylation. MSL1 expression correlates with immune infiltration across cancers, and its deletion markedly reduces CD274 expression. Mechanistically, MSL1 regulates CD274-associated genes, including BIRC3 and HLA-A, and may act competitively with STAT3 in controlling CD274 transcription. Together, these findings identify MSL1 as a key epigenetic regulator of tumor immune evasion and highlight it as a potential therapeutic target for enhancing cancer immunotherapy efficacy.

## Data Availability

The H4K16ac ChIP sequencing dataset presented in the study are deposited in the GEO repository, accession number GSE198645.
