# Correction: Epigenetic activation of CD274/PD-L1 by the MSL complex expands its role beyond dosage compensation

**DOI:** 10.3389/fimmu.2025.1761945

**Published:** 2025-12-19

**Authors:** Aiping Wen, Xuanfei Feng, Yingying Li, Xueli Cui, Qixian Zou, Yong Cai, Jingji Jin, Yunxiao He

**Affiliations:** 1 Department of Gynecology and Obstetrics, Affiliated Hospital of North Sichuan Medical College, Nanchong, Sichuan, China; 2 Changchun GeneScience Pharmaceutical Co., Ltd., Changchun, Jilin, China; 3 School of Life Sciences, Jilin University, Changchun, Jilin, China

**Keywords:** histone acetyltransferase, CD274, transcriptional regulation, male-specific lethal, H4K16Ac

There was a mistake in the caption of **Figure 6** as published. During the revision of the manuscript, we supplemented co-immunoprecipitation assays to confirm the physical interaction between MSL1 and MOF. We transfected a Flag-MOF-tagged plasmid into 293T cells; however, during figure preparation, we mistakenly labeled Lane 5 in **Figure 6E** as “Flag-MOFK274A”, which should be corrected to “Flag-MOF”. Following the co-immunoprecipitation assays, we used antibodies against the Flag tag and CD274, respectively. In the Western blot annotation in **Figure 6E**, we erroneously wrote “IB: GAPDH”, which should be changed to “IB: CD274”. This correction can be verified by the marker positions in the original Western blot images provided in our supplementary materials. The corrected caption of **Figure 6** appears below.

Also, there was a mistake in the caption of **Figure 5** as published. During the revision of the manuscript, we performed grayscale quantification of the Western blot images as requested by the reviewers. However, in **Figure 5B**, the IB: GAPDH band was not properly aligned with the corresponding protein blot shown below it. The corrected caption of **Figure 5** appears below.

**Figure 5:**
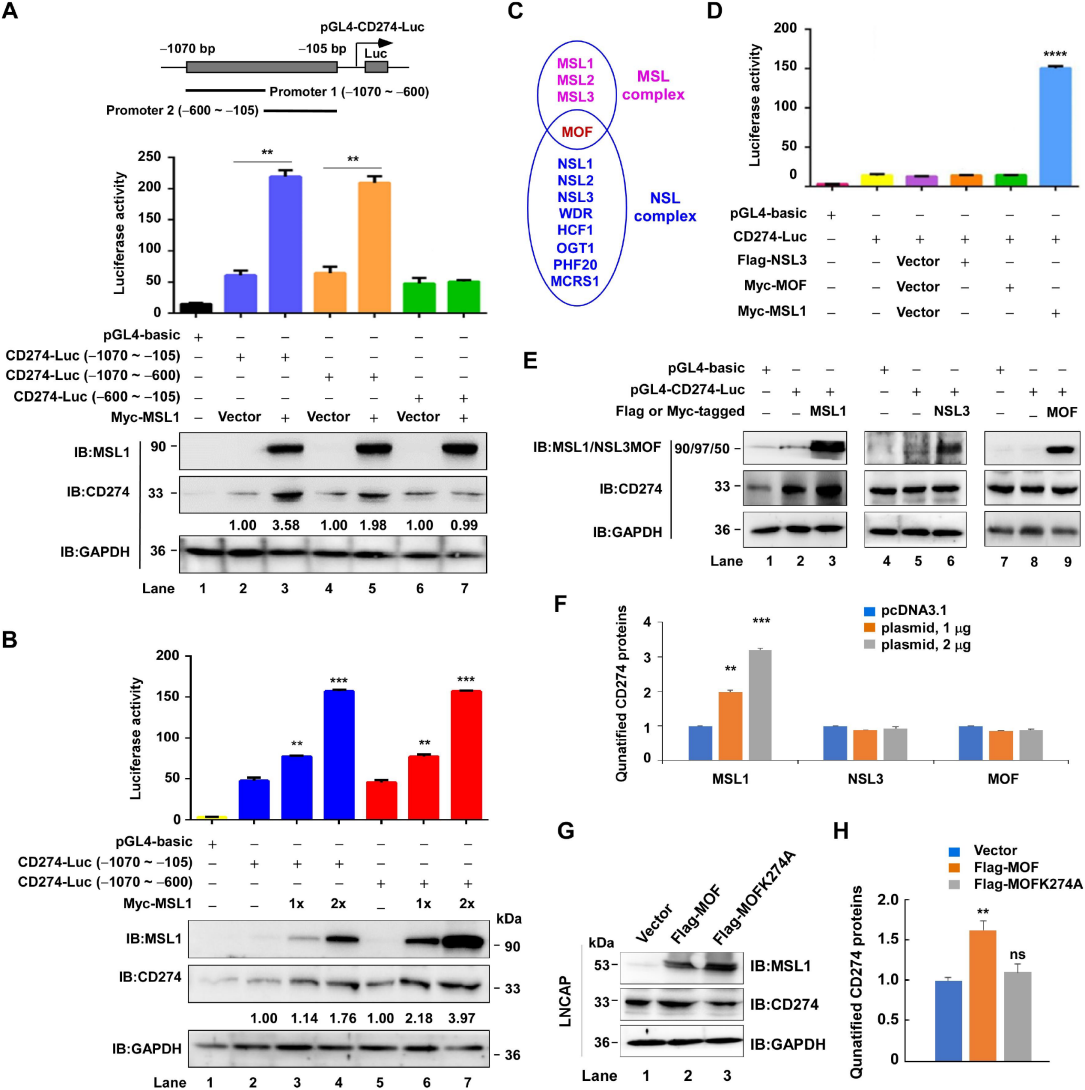
MSL1 transcriptionally regulates CD274 promoter activity. **(A)** Dual-luciferase reporter and Western blot analysis demonstrating the transcriptional regulation of CD274 by MSL1. Schematic shows luciferase reporter constructs containing CD274 promoter fragments (−1070 to −105 bp, −1070 to −600 bp, and −600 to −105bp) fused to firefly luciferase (Luc). **(B)** Luciferase reporter and Western blot analysis of CD274 promoter activity in cells co-transfected with pGL4-basic or pGL4-Luc constructs containing the proximal (−1070 to −105 bp) or distal (−1070 to −600 bp) CD274 promoter regions, together with increasing doses of Myc-MSL1 expression vector (1× or 2×). **(C)** Schematic illustration of MOF within two distinct complexes: MSL (left) and NSL (right). **(D)** Luciferase reporter assay assessing the impact of NLS3, MOF and MSL1 on CD274 promoter activation. Cells were co-transfected with the indicated plasmid combinations. **(E, F)**. Western blot analysis **(E)** and quantification **(F)** of CD274 protein levels in HEK293T cells following transfection with varying doses of Flag- or Myc-tagged MSL1, NSL3, or MOF. **(G, H)**. Western blot analysis **(G)** and quantification **(H)** of CD274 protein levels in LNCaP cells following transfection with Flag-MOF or Flag-MOFK274A plasmid. Statistical significance: **p < 0.01; ***p < 0.001; ****p < 0.0001. Data are presented as mean ± SEM from three independent experiments (n=3). GAPDH was used as the internal control in all Western blot analyses.

**Figure 6:**
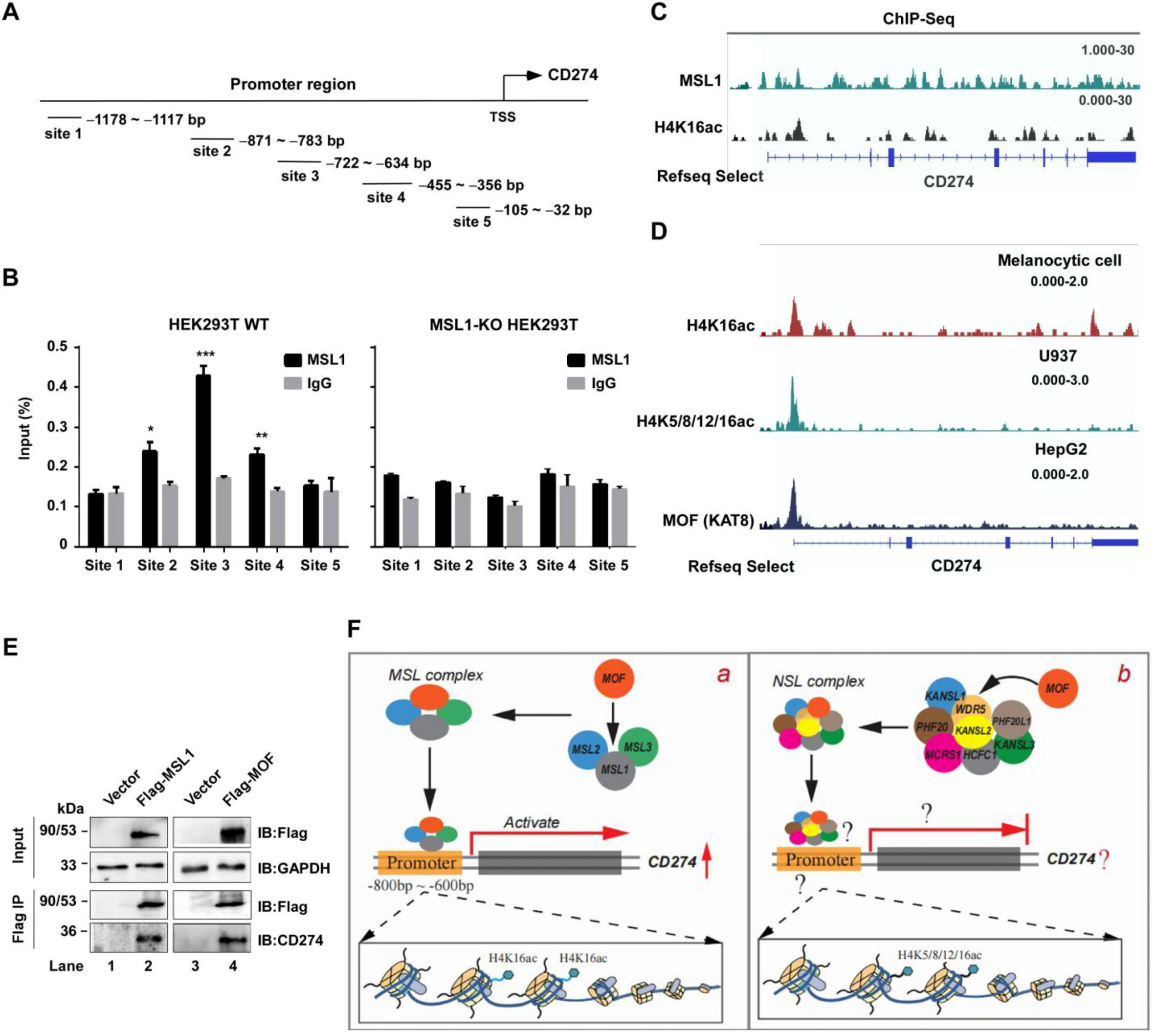
MSL1 binding and epigenetic regulation of the CD274 promoter. **(A)** CD274 promoter architecture. Schematic representation of the CD274 promoter region highlighting five putative binding sites. **(B)** MSL1 occupancy at the CD274 promoter. ChIP–qPCR analysis of MSL1 enrichment across the CD274 promoter in HEK293T cells. The left panel shows wild-type (WT) cells, whereas the right panel depicts MSL1- KO cells. Data are shows as mean ± SD from six independent experiments (n=6). Statistical significance: p < 0.05; p < 0.01; p < 0.001. **(C)** Epigenetic landscape of the CD274 locus showing MSL1 and H4K16ac enrichment in HEK293T cells. **(D)** Epigenetic landscape of the CD274 locus. IGV snapshots showing histone acetylation and MOF enrichment across the CD274 gene region in Melanocytic cells (H4K16ac, top), U937 cells (H4K5/8/12/16ac, middle), and HepG2 cells (KAT8/MOF, bottom). **(E)** Co-immunoprecipitation assays were performed in HEK293T cells using Flag-MSL1 and Flag-MOF constructs with an anti-Flag antibody. GAPDH was used as the internal control in all Western blot analyses. **(F)** Model of MSL and NSL complex–mediated regulation. Proposed mechanisms of CD274 transcriptional regulation. (a) The MSL complex activates CD274 transcription by recruiting MSL1 to the −800 to −600 bp region of the promoter, where MOF acetylates H4K16. (b) The NSL complex may also target the CD274 promoter through an unidentified subunit, potentially involving histone modifications such as H4K5/8/12/16ac. Gashed lines and question marks denote hypothetical or unresolved mechanisms.

The original version of this article has been updated.

