# Supplementary material for: Epigenetic activation of CD274/PD-L1 by the MSL complex expands its role beyond dosage compensation
Source: Front Immunol. 2025 Dec 2;16:1711451. doi: 10.3389/fimmu.2025.1711451 (PMC12705358; doi:10.3389/fimmu.2025.1711451)
Supplement: Supplementary file 1 [file DataSheet1.pdf]

Figure 2B

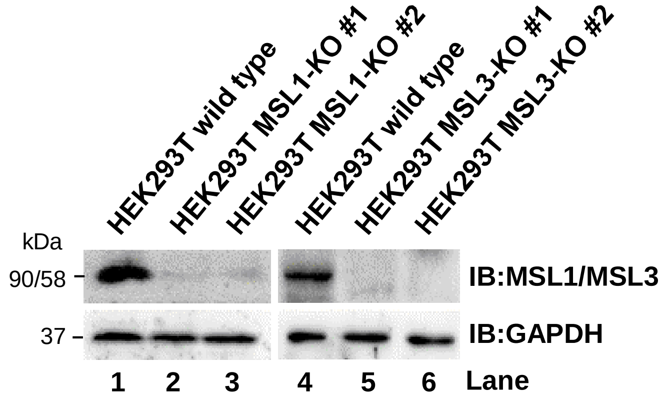
HEK293T wild type
HEK293T MSL1-KO #1
HEK293T MSL1-KO #2
HEK293T wild type
HEK293T MSL3-KO #1
HEK293T MSL3-KO #2
kDa
90/58
37
IB:MSL1/MSL3
IB:GAPDH
1
2
3
4
5
6
Lane

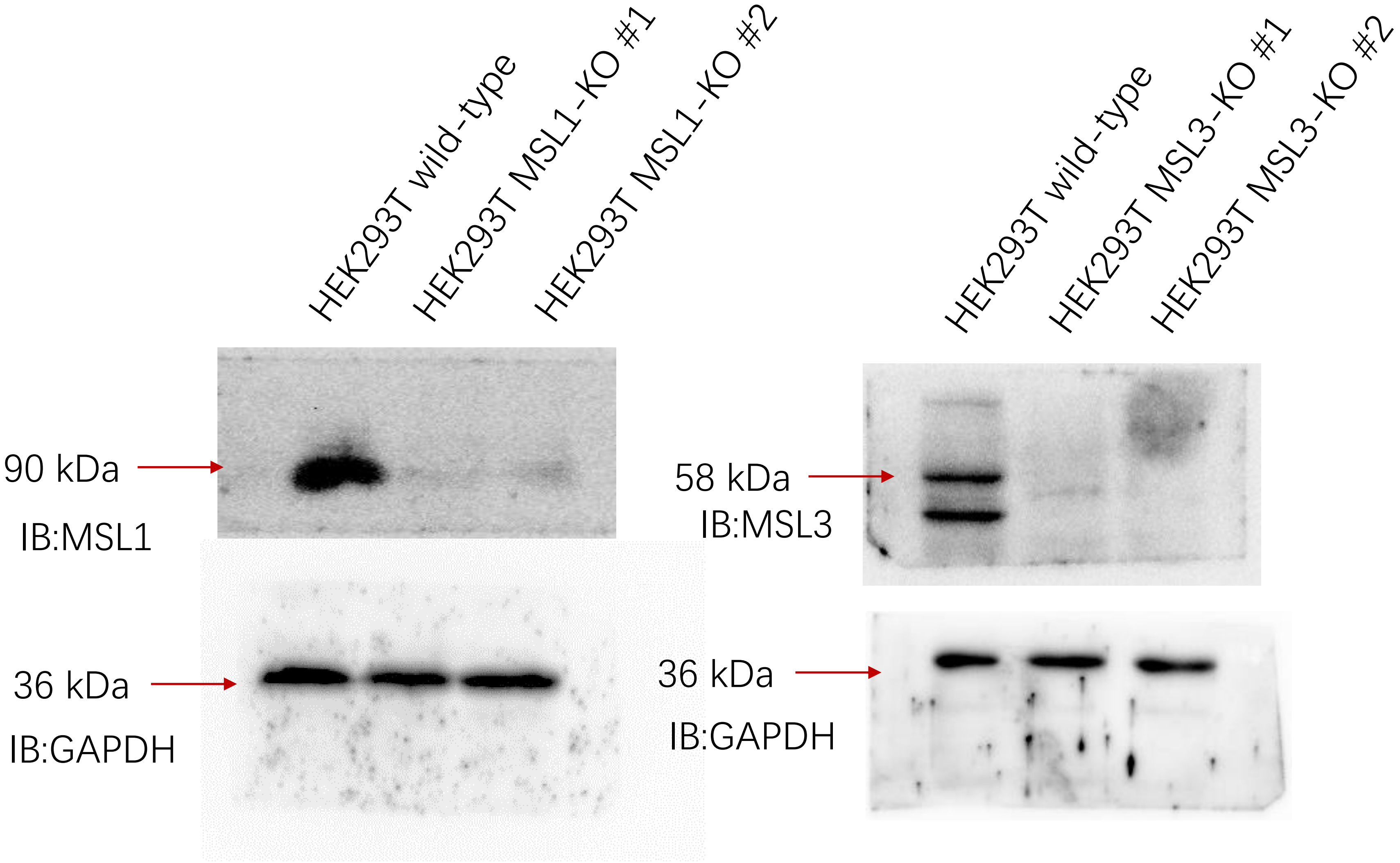
HEK293T wild-type
HEK293T MSL1-KO #1
HEK293T MSL1-KO #2
HEK293T wild-type
HEK293T MSL3-KO #1
HEK293T MSL3-KO #2
90 kDa
IB:MSL1
36 kDa
IB:GAPDH
58 kDa
IB:MSL3
36 kDa
IB:GAPDH

Figure 3D

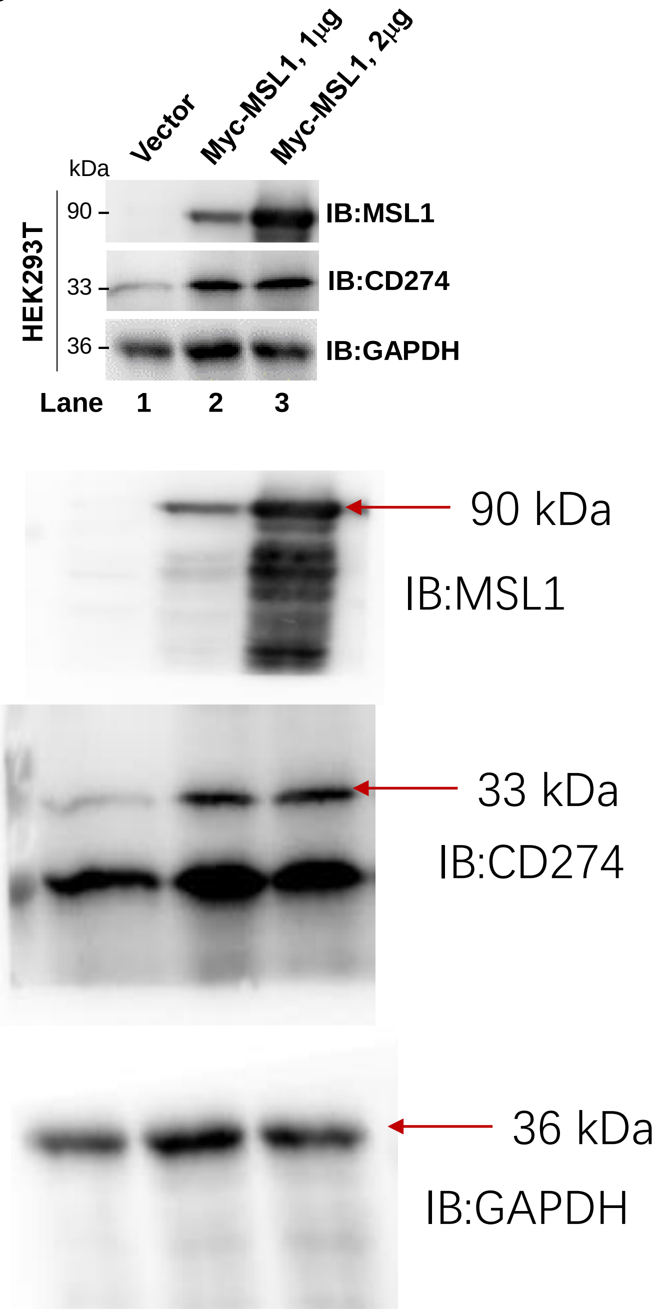

Figure 3E

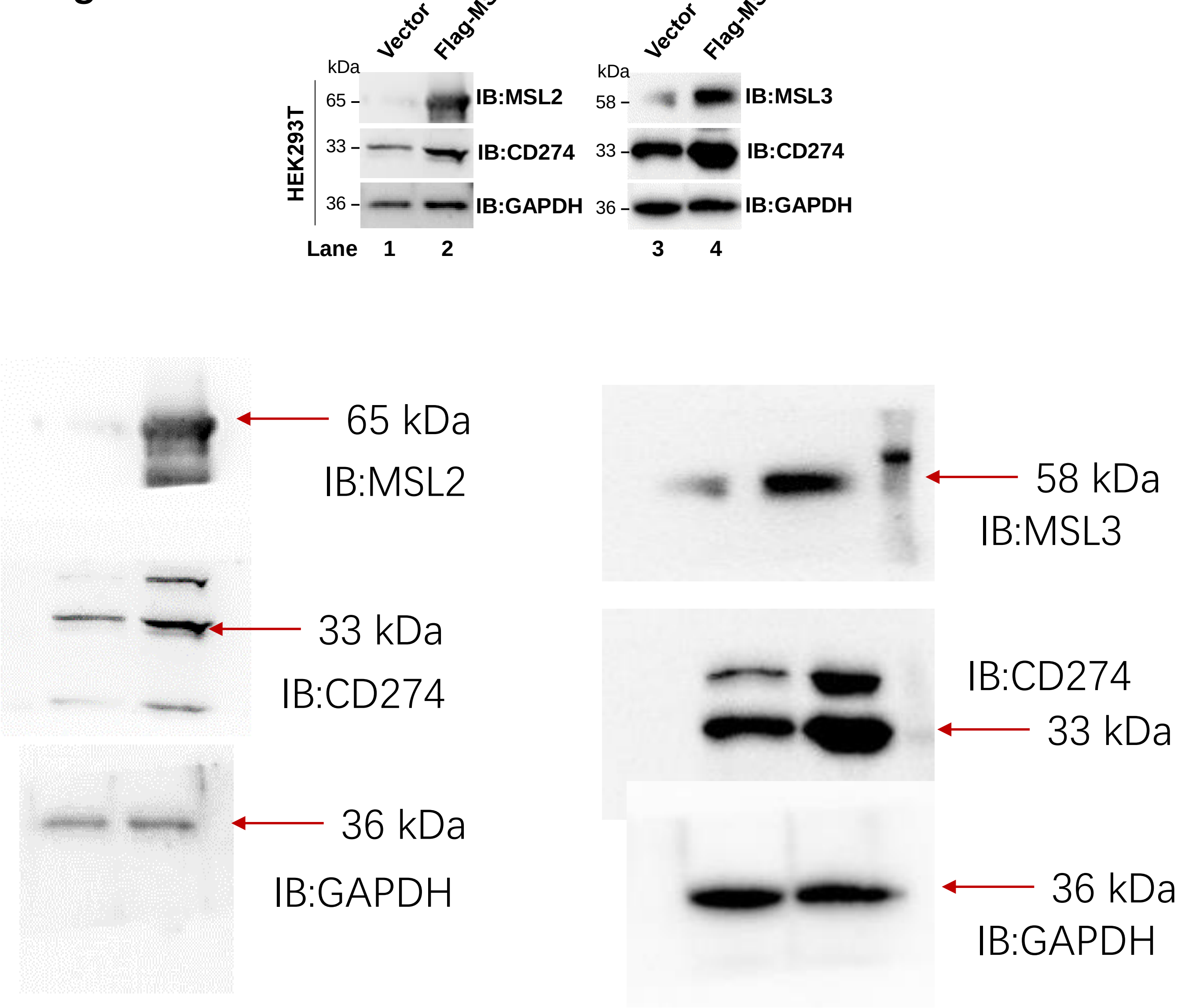

Figure 3G

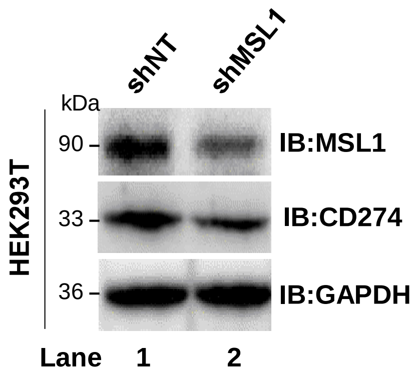
shNT
shMSL1
kDa
90
33
36
HEK293T
IB:MSL1
IB:CD274
IB:GAPDH
Lane
1
2

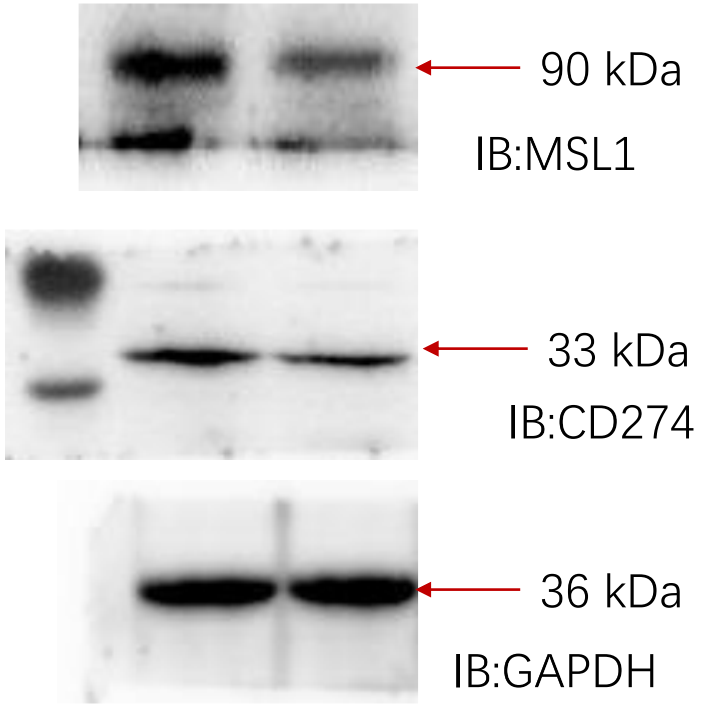
90 kDa
IB:MSL1
33 kDa
IB:CD274
36 kDa
IB:GAPDH

Figure 3H

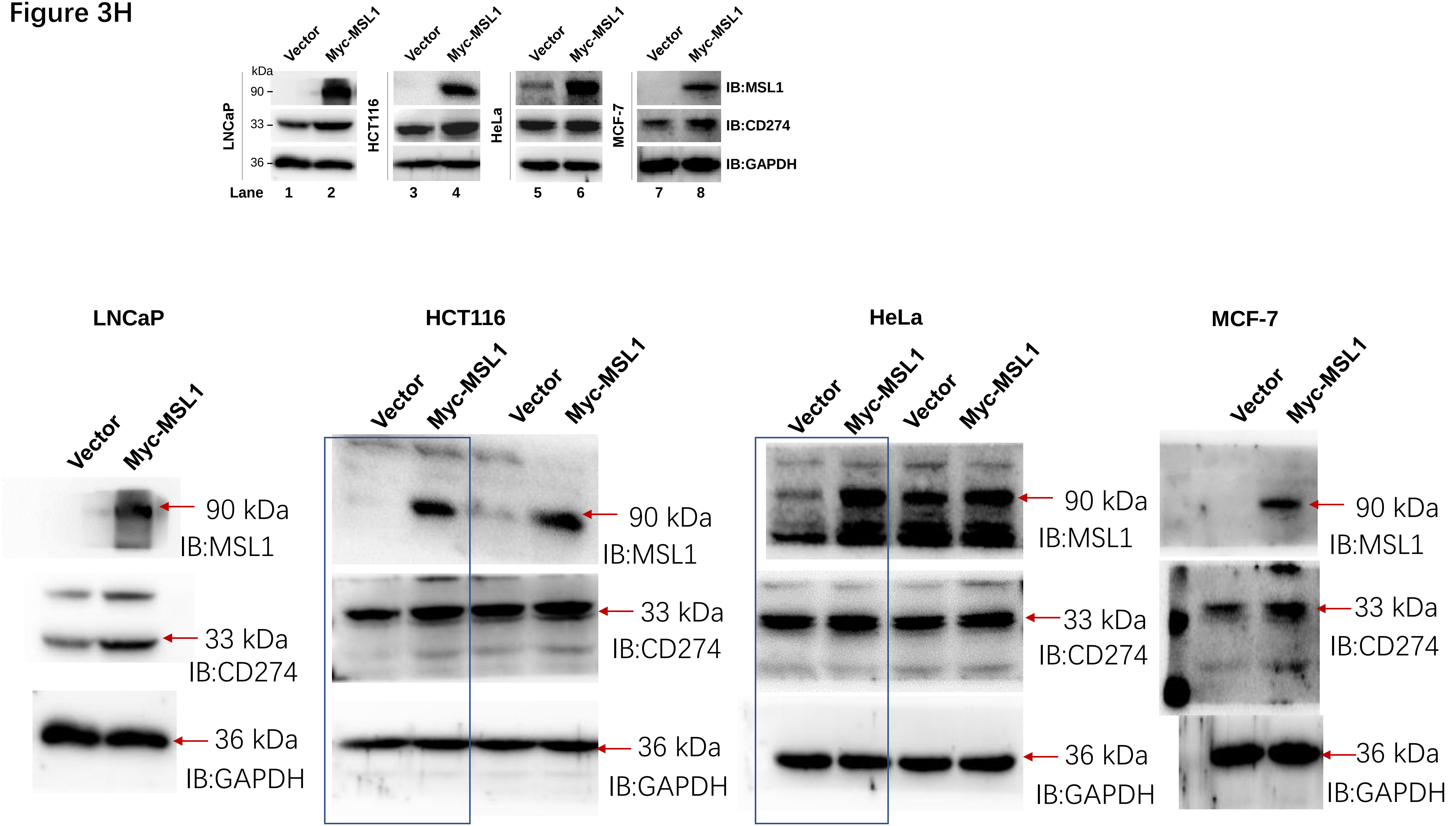
Vector
Myc-MSL1
kDa
90
33
36
LNCaP
HCT116
HeLa
MCF-7
IB:MSL1
IB:CD274
IB:GAPDH
Lane
1
2
3
4
5
6
7
8
LNCaP
HCT116
HeLa
MCF-7
90 kDa
33 kDa
36 kDa

Figure 3J

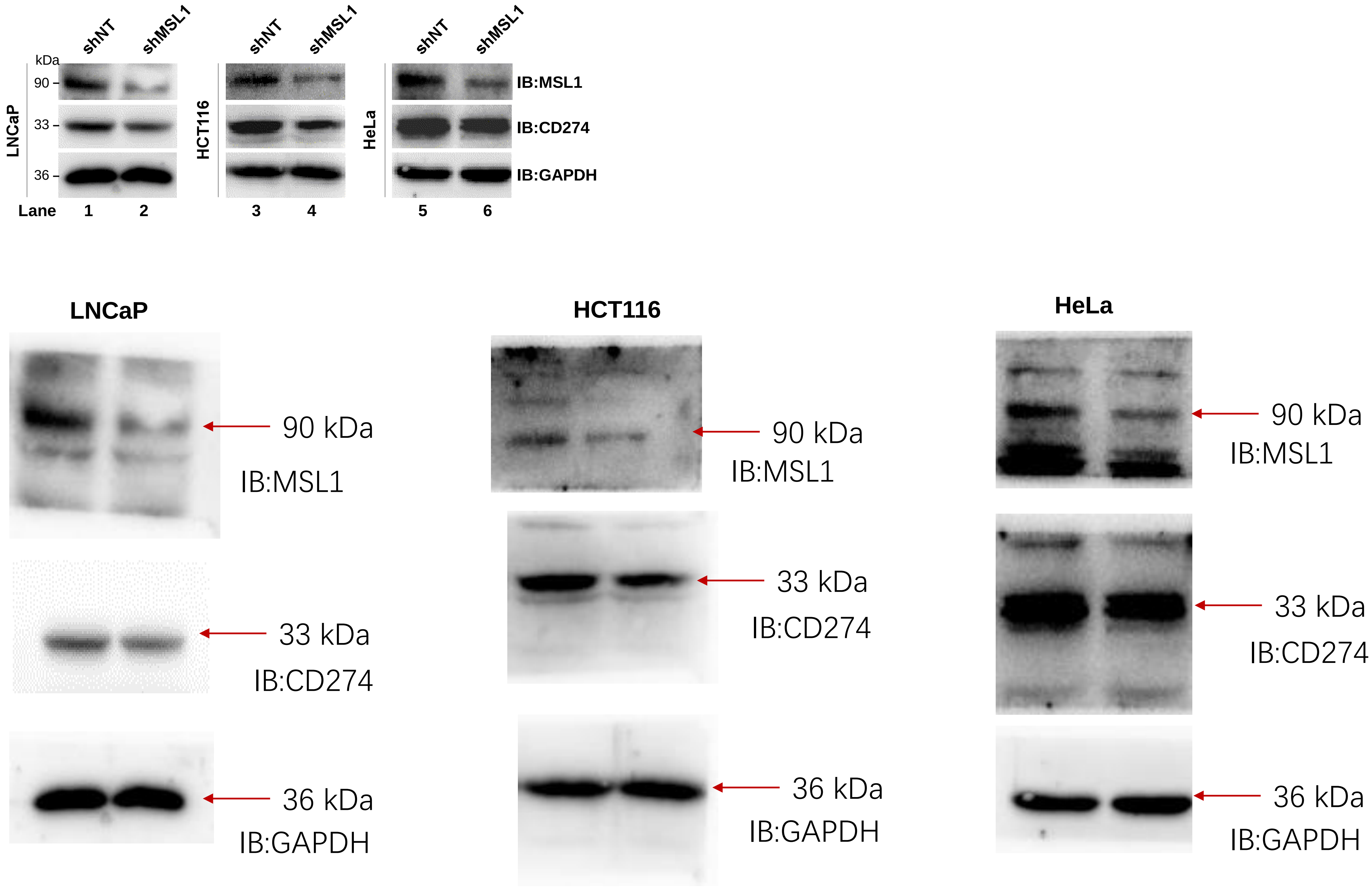

shNT
shMSL1
kDa
90
33
36
LNCaP
HCT116
HeLa
IB:MSL1
IB:CD274
IB:GAPDH
Lane
1
2
3
4
5
6
LNCaP
HCT116
HeLa
90 kDa
IB:MSL1
33 kDa
IB:CD274
36 kDa
IB:GAPDH

Figure 4A

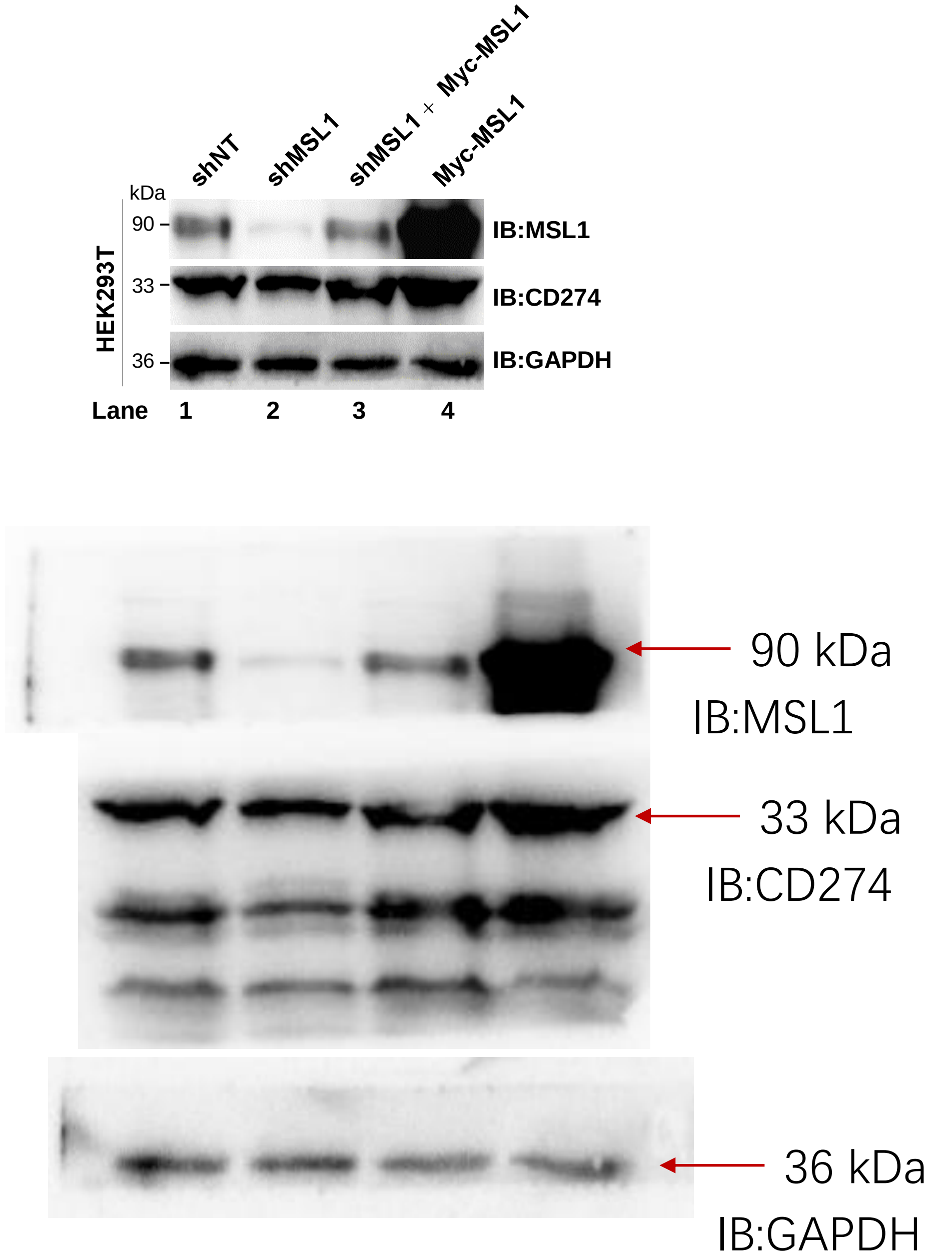

Figure 4C

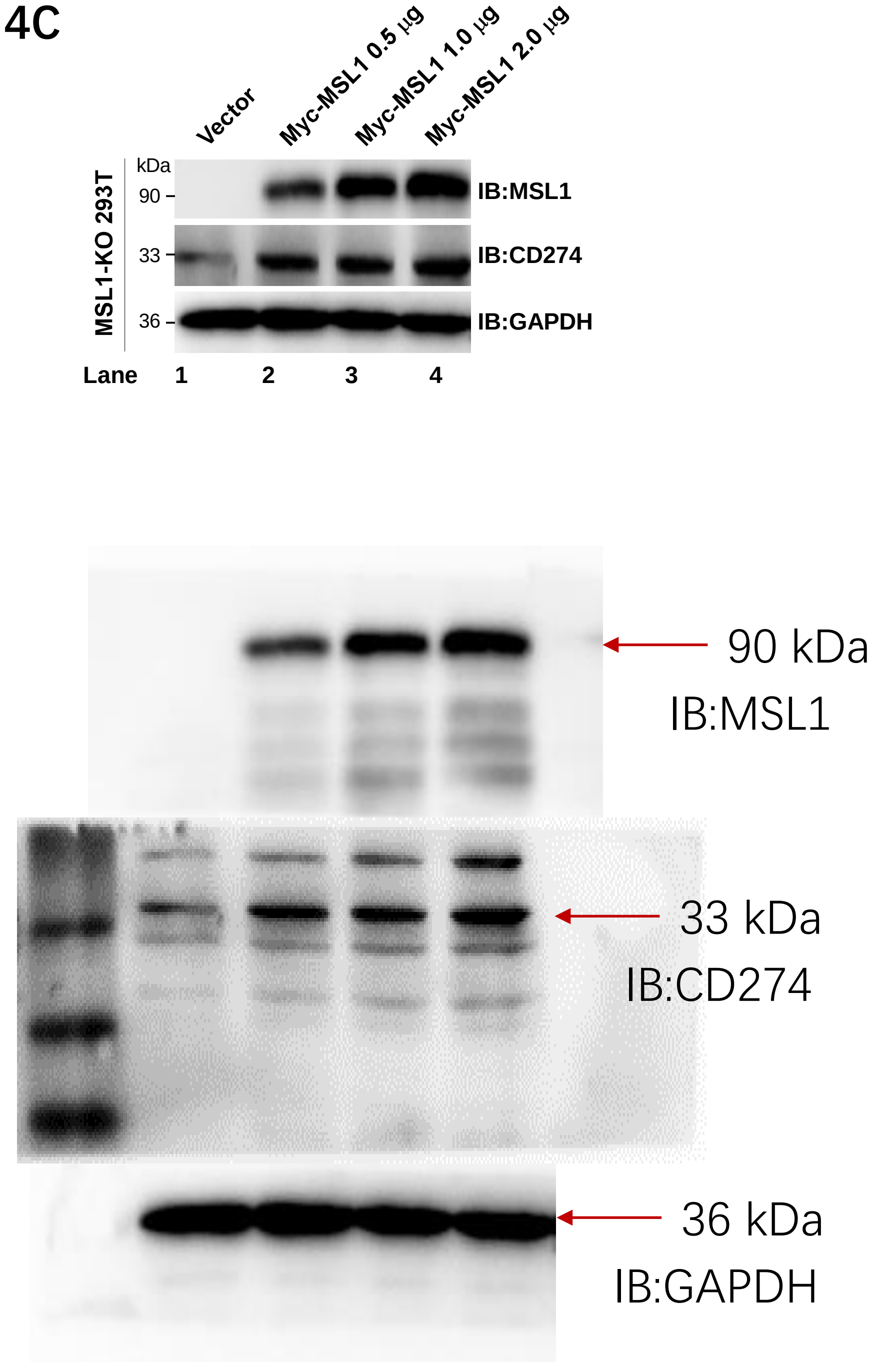

Figure 4G

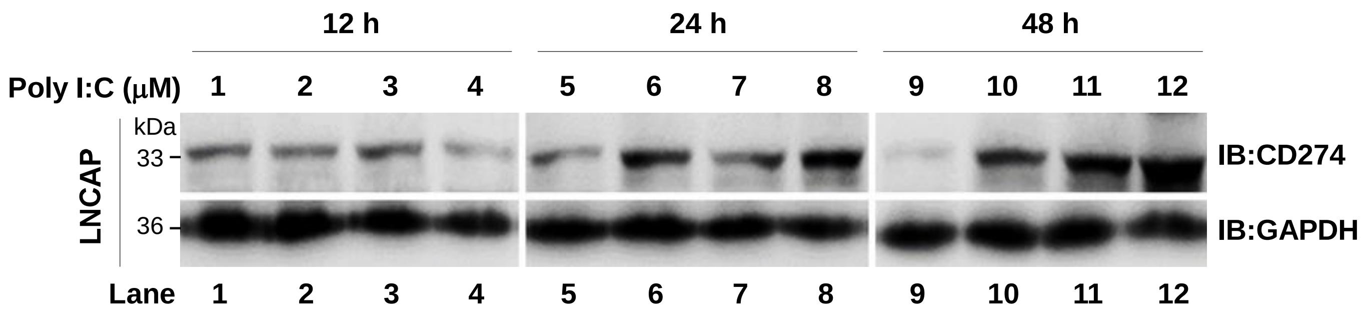

12 h
24 h
48 h
Poly I:C (μM) 1 2 3 4 5 6 7 8 9 10 11 12
kDa
33
36
LNCAP
IB:CD274
IB:GAPDH
Lane 1 2 3 4 5 6 7 8 9 10 11 12

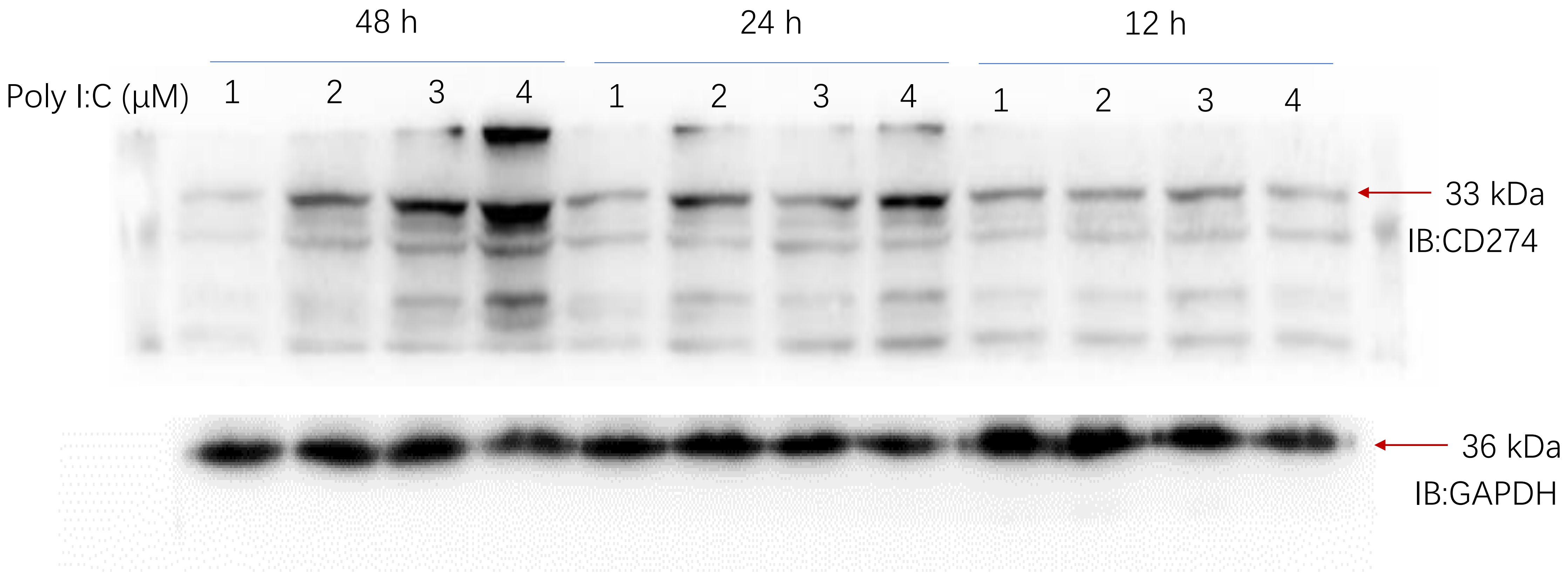

48 h
24 h
12 h
Poly I:C (μM) 1 2 3 4 1 2 3 4 1 2 3 4
33 kDa
IB:CD274
36 kDa
IB:GAPDH

Figure 4I

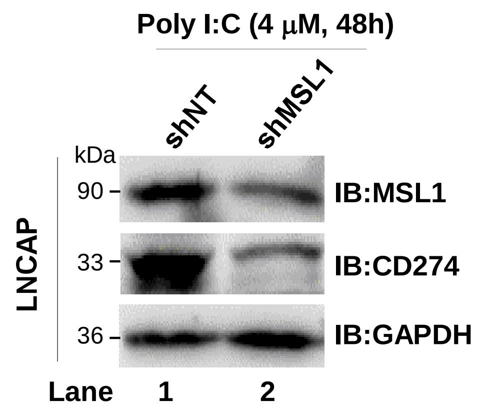

Poly I:C (4 μM, 48h)
shNT
shMSL1
kDa
90
33
36
LNCAP
IB:MSL1
IB:CD274
IB:GAPDH
Lane
1
2

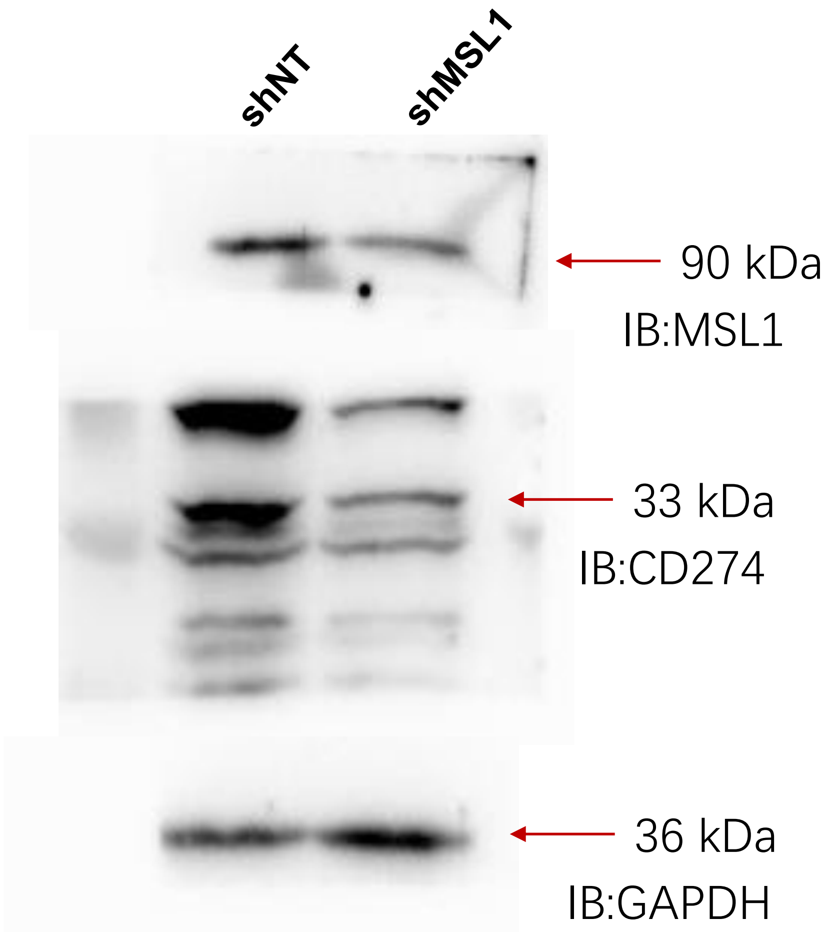

shNT
shMSL1
90 kDa
IB:MSL1
33 kDa
IB:CD274
36 kDa
IB:GAPDH

Figure 5A

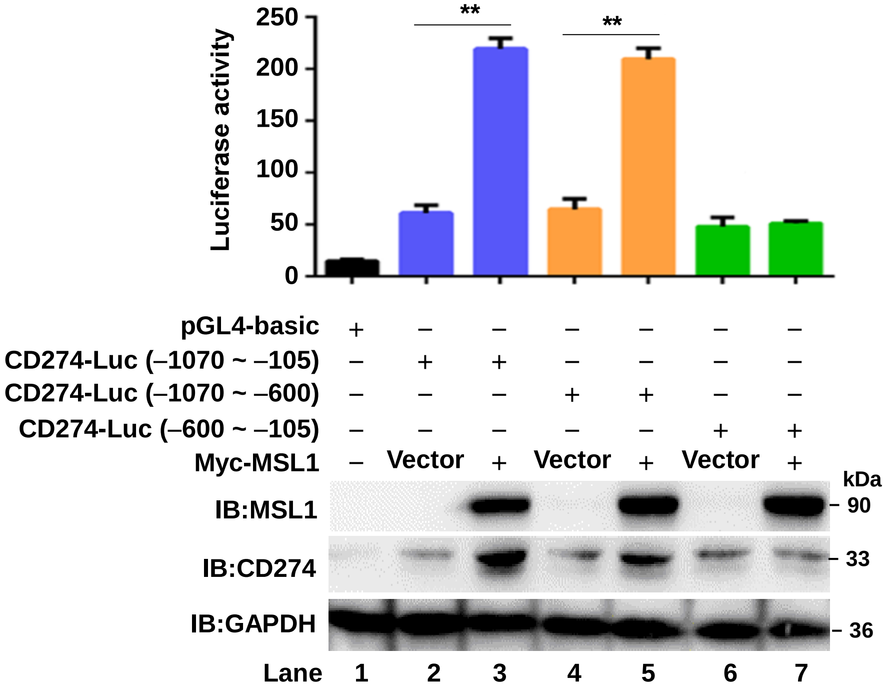

Luciferase activity
250
200
150
100
50
0
**
**
pGL4-basic + – – – – – –
CD274-Luc (–1070 ~ –105) – + + – – – –
CD274-Luc (–1070 ~ –600) – – – + + – –
CD274-Luc (–600 ~ –105) – – – – – + +
Myc-MSL1 – Vector + Vector + Vector +
IB:MSL1
IB:CD274
IB:GAPDH
kDa
90
33
36
Lane 1 2 3 4 5 6 7

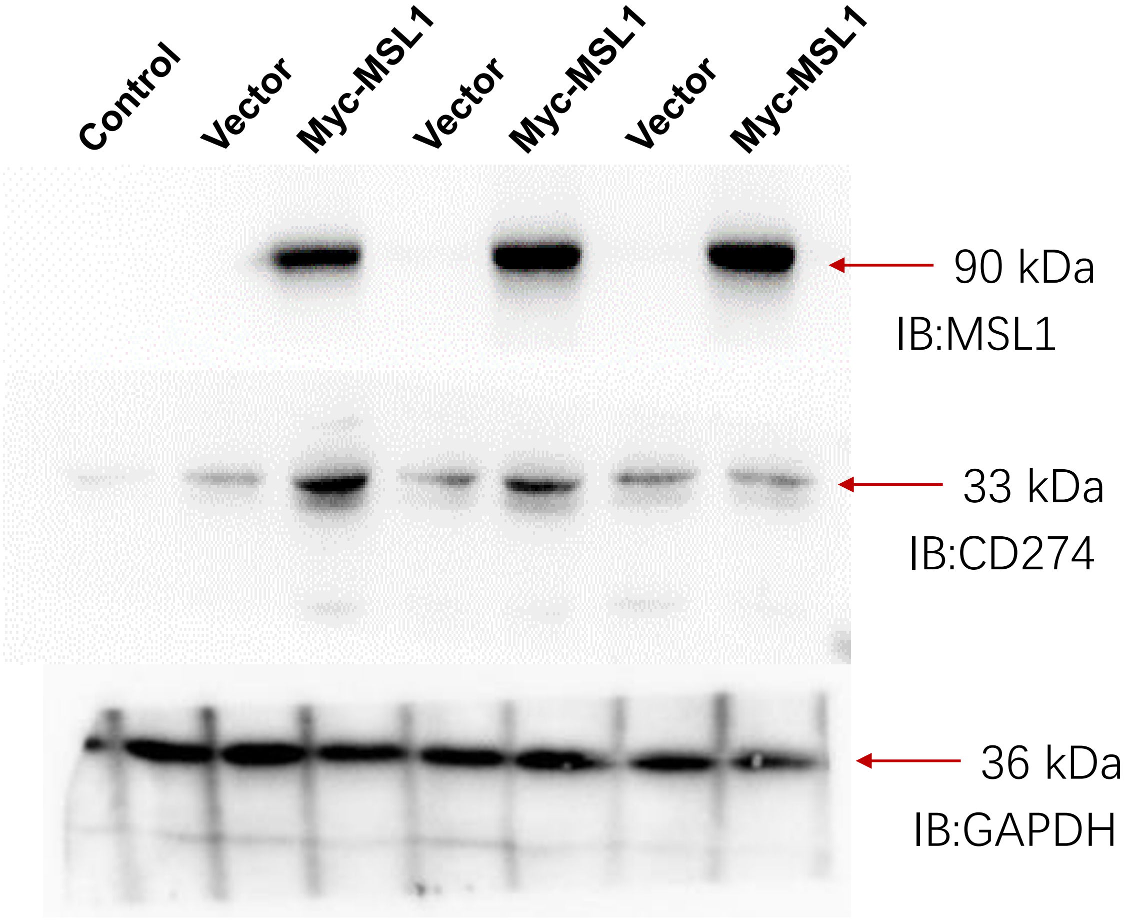

Control
Vector
Myc-MSL1
Vector
Myc-MSL1
Vector
Myc-MSL1
90 kDa
IB:MSL1
33 kDa
IB:CD274
36 kDa
IB:GAPDH

Figure 5B

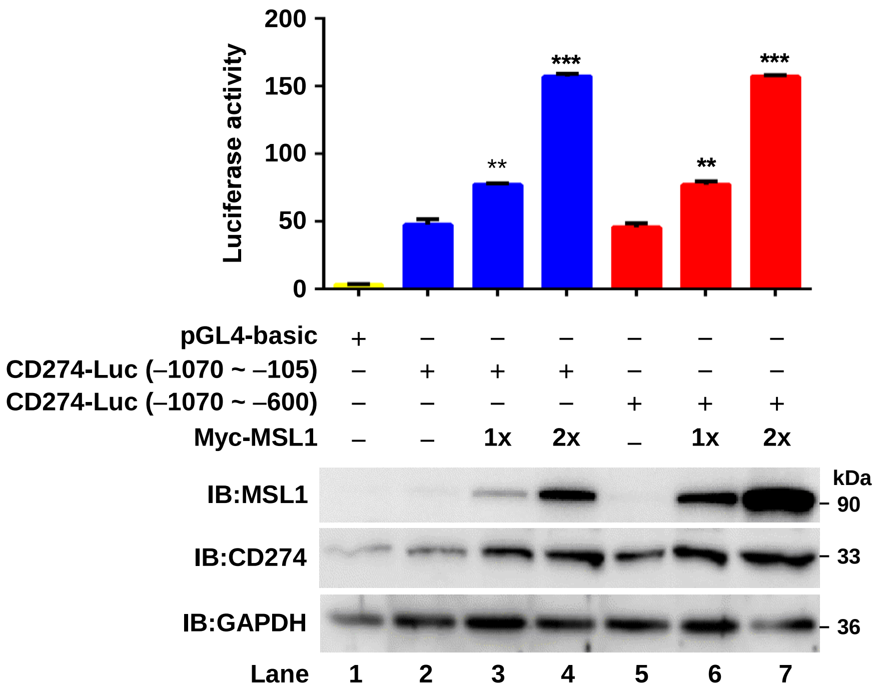

Luciferase activity
200
150
100
50
0
**
***
**
***
pGL4-basic + – – – – – –
CD274-Luc (–1070 ~ –105) – + + + – – –
CD274-Luc (–1070 ~ –600) – – – – + + +
Myc-MSL1 – – 1x 2x – 1x 2x
IB:MSL1
IB:CD274
IB:GAPDH
kDa
90
33
36
Lane 1 2 3 4 5 6 7

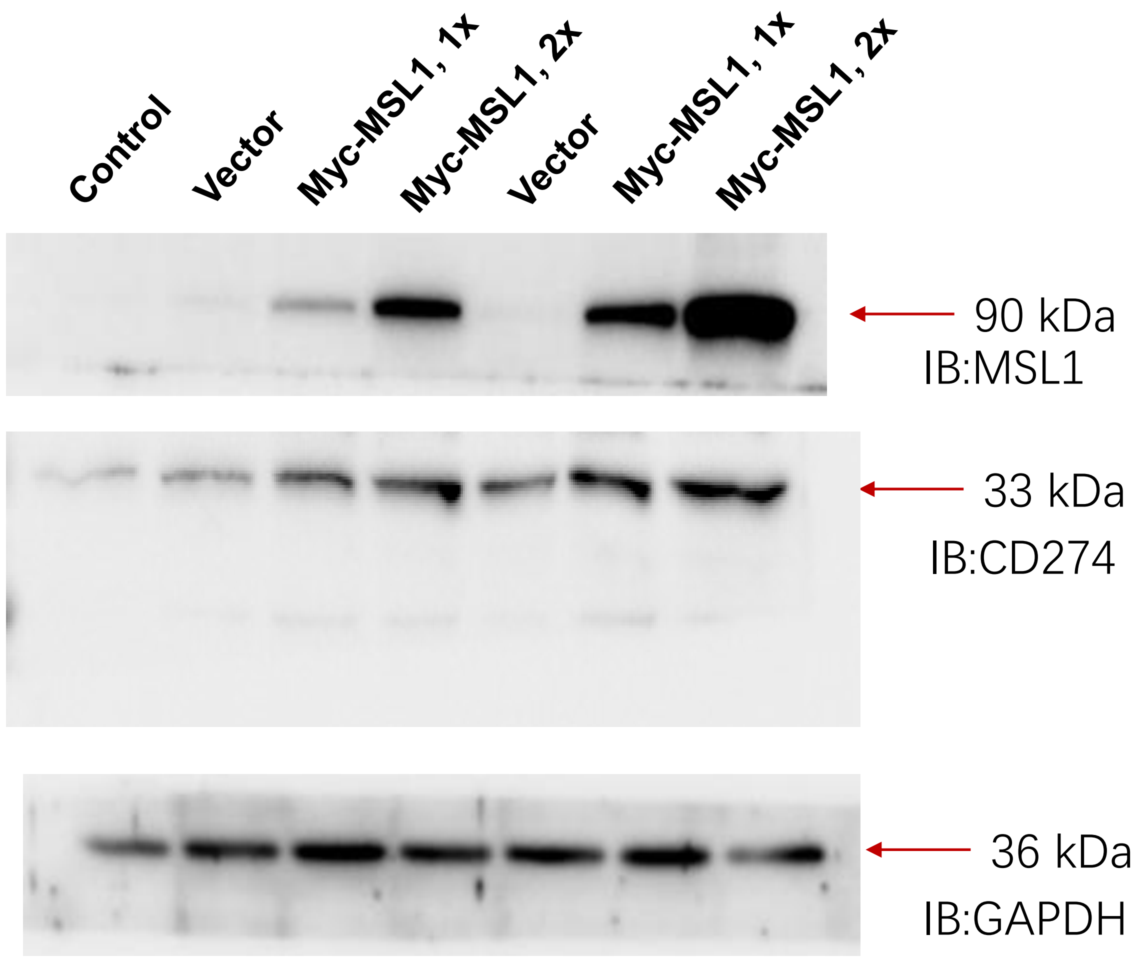

Control
Vector
Myc-MSL1, 1x
Myc-MSL1, 2x
Vector
Myc-MSL1, 1x
Myc-MSL1, 2x
90 kDa
IB:MSL1
33 kDa
IB:CD274
36 kDa
IB:GAPDH

Figure 5E

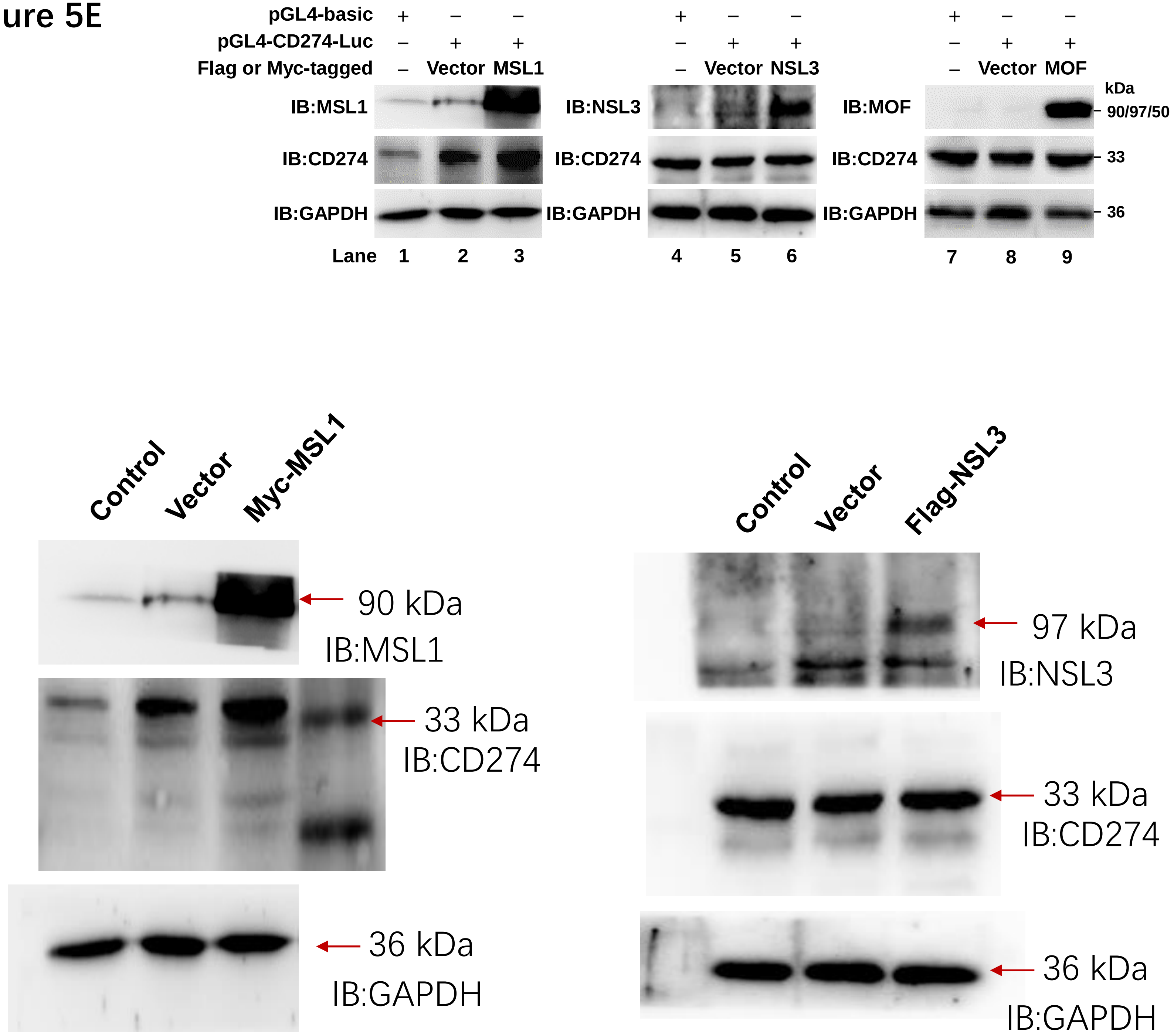

pGL4-basic + – – + – – + – –
pGL4-CD274-Luc – + + – + + – + +
Flag or Myc-tagged – Vector MSL1 – Vector NSL3 – Vector MOF
kDa
IB:MSL1
IB:NSL3
IB:MOF
90/97/50
IB:CD274
33
IB:GAPDH
36
Lane 1 2 3 4 5 6 7 8 9
Control
Vector
Myc-MSL1
90 kDa
IB:MSL1
33 kDa
IB:CD274
36 kDa
IB:GAPDH
Flag-NSL3
97 kDa
IB:NSL3
Myc-MOF
53 kDa
IB:MOF

Figure 5G

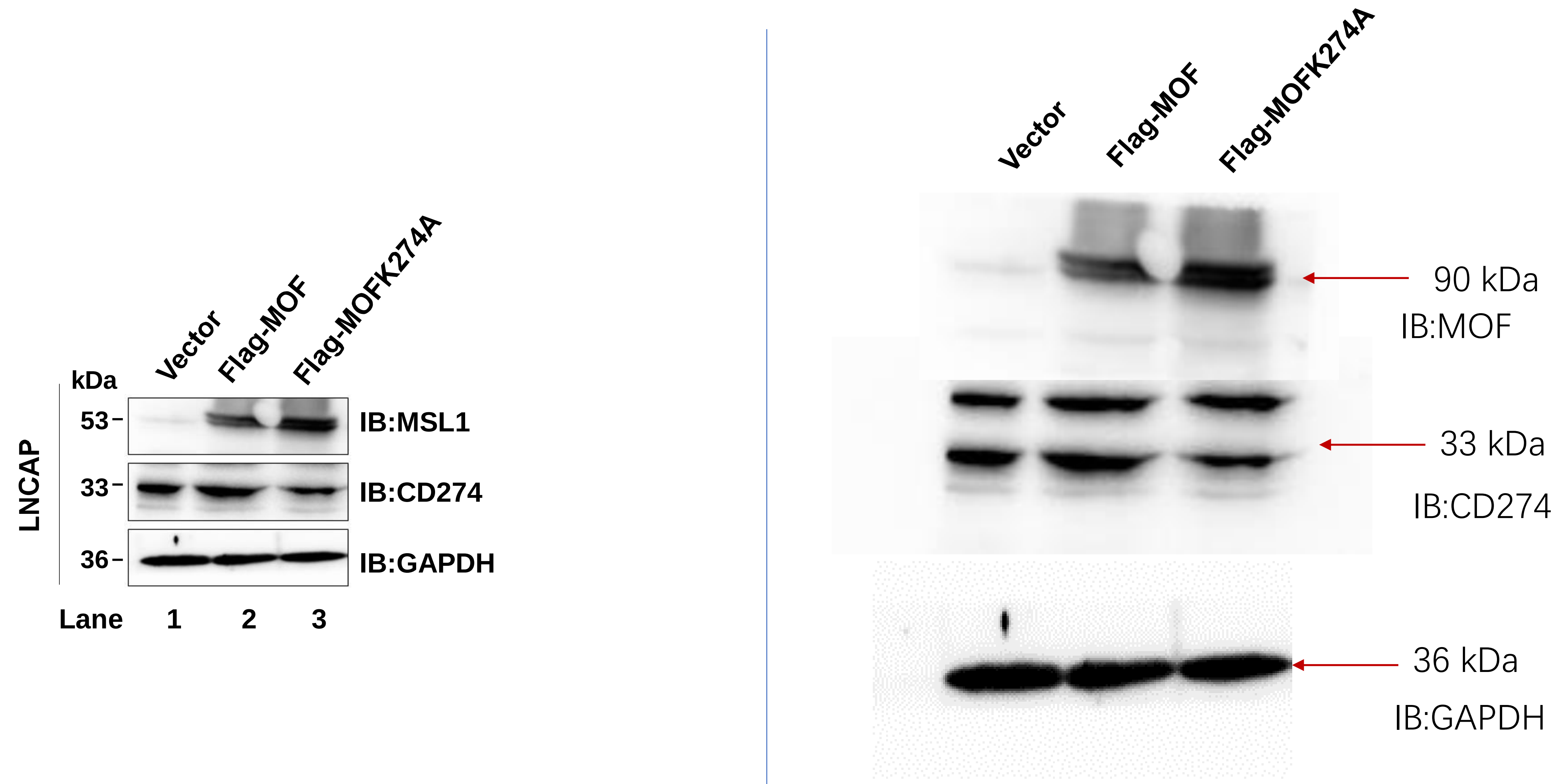
Vector
Flag-MOF
Flag-MOFK274A
kDa
53
33
36
LNCAP
IB:MSL1
IB:CD274
IB:GAPDH
Lane 1 2 3
Vector
Flag-MOF
Flag-MOFK274A
90 kDa
IB:MOF
33 kDa
IB:CD274
36 kDa
IB:GAPDH

Figure 6E

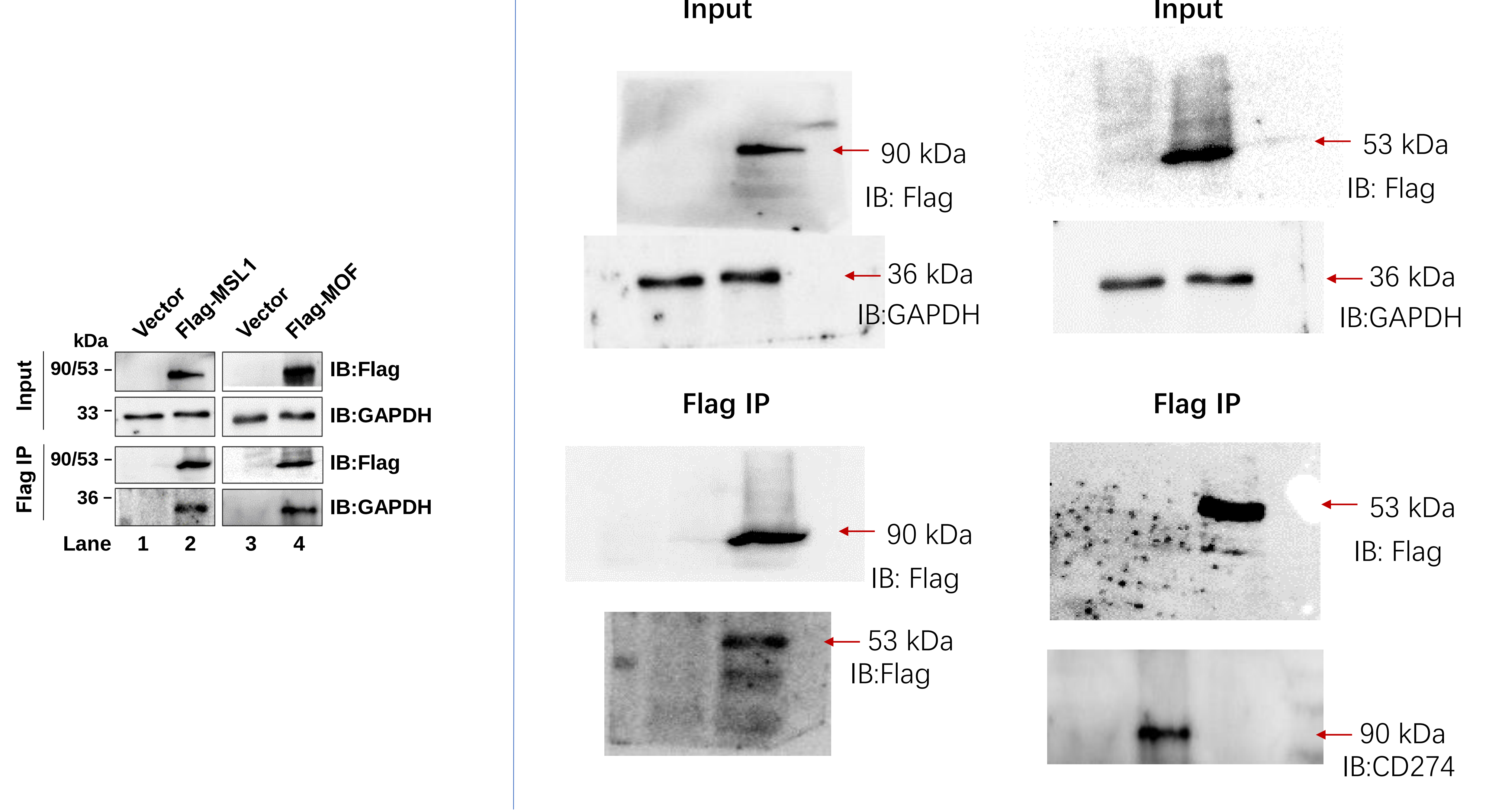
Vector
Flag-MSL1
Vector
Flag-MOF
kDa
Input
Flag IP
90/53
33
90/53
36
IB:Flag
IB:GAPDH
IB:Flag
IB:GAPDH
Lane 1 2 3 4
Input
90 kDa
IB: Flag
36 kDa
IB:GAPDH
Input
53 kDa
IB: Flag
36 kDa
IB:GAPDH
Flag IP
90 kDa
IB: Flag
53 kDa
IB:Flag
Flag IP
53 kDa
IB: Flag
90 kDa
IB:CD274
